# Comparative Study on the Performance of Three Detection Methods for the Quantification of Pacific Ciguatoxins in French Polynesian Strains of *Gambierdiscus polynesiensis*

**DOI:** 10.3390/md20060348

**Published:** 2022-05-25

**Authors:** Hélène Taiana Darius, Taina Revel, Jérôme Viallon, Manoëlla Sibat, Philippe Cruchet, Sébastien Longo, Donnie Ransom Hardison, William C. Holland, Patricia A. Tester, R. Wayne Litaker, Jennifer R. McCall, Philipp Hess, Mireille Chinain

**Affiliations:** 1 Institut Louis Malardé (ILM), Laboratory of Marine Biotoxins, UMR 241-EIO (IFREMER, ILM, IRD, Université de Polynésie Française), P.O. Box 30, Papeete 98713, French Polynesia; trevel@ilm.pf (T.R.); jviallon@ilm.pf (J.V.); pcruchet@ilm.pf (P.C.); sebastien.longo81@gmail.com (S.L.); mchinain@ilm.pf (M.C.); 2 IFREMER, PHYTOX, Laboratoire METALG, F-44000 Nantes, France; manoella.sibat@ifremer.fr (M.S.); philipp.hess@ifremer.fr (P.H.); 3 National Oceanic and Atmospheric Administration, Center for Coastal Fisheries and Habitat Research, Beaufort, NC 28516, USA; rance.hardison@noaa.gov (D.R.H.); chris.holland@noaa.gov (W.C.H.); 4 Ocean Tester, LLC, Beaufort, NC 28516, USA; ocean.tester@gmail.com; 5 CSS, Inc. Under Contract to National Oceanic and Atmospheric Administration, National Centers for Coastal Ocean Science, National Ocean Service, Beaufort, NC 28516, USA; wayne.r.litaker@noaa.gov; 6 Center for Marine Science, University of North Carolina Wilmington, 601 South College Road, Wilmington, NC 28403, USA; mccalljr@uncw.edu

**Keywords:** ciguatera poisoning, *Gambierdiscus polynesiensis*, ciguatoxins, fluorescent receptor binding assay, neuroblastoma cell-based assay, liquid chromatography tandem mass spectrometry, risk assessment

## Abstract

*Gambierdiscus* and *Fukuyoa* dinoflagellates produce a suite of secondary metabolites, including ciguatoxins (CTXs), which bioaccumulate and are further biotransformed in fish and marine invertebrates, causing ciguatera poisoning when consumed by humans. This study is the first to compare the performance of the fluorescent receptor binding assay (fRBA), neuroblastoma cell-based assay (CBA-N2a), and liquid chromatography tandem mass spectrometry (LC-MS/MS) for the quantitative estimation of CTX contents in 30 samples, obtained from four French Polynesian strains of *Gambierdiscus polynesiensis*. fRBA was applied to *Gambierdiscus* matrix for the first time, and several parameters of the fRBA protocol were refined. Following liquid/liquid partitioning to separate CTXs from other algal compounds, the variability of CTX contents was estimated using these three methods in three independent experiments. All three assays were significantly correlated with each other, with the highest correlation coefficient (*r*^2^ = 0.841) found between fRBA and LC-MS/MS. The CBA-N2a was more sensitive than LC-MS/MS and fRBA, with all assays showing good repeatability. The combined use of fRBA and/or CBA-N2a for screening purposes and LC-MS/MS for confirmation purposes allows for efficient CTX evaluation in *Gambierdiscus*. These findings, which support future collaborative studies for the inter-laboratory validation of CTX detection methods, will help improve ciguatera risk assessment and management.

## 1. Introduction

The dinoflagellates responsible for ciguatera poisoning (CP) belong to a benthic microalgal genus known as *Gambierdiscus*, identified for the first time as *G. toxicus* in the Gambier Archipelago (French Polynesia) [1]. With the help of morphological characteristics and phylogenetic analyses, 17 other *Gambierdiscus* species have been identified worldwide: *G. belizeanus*, *G. australes*, *G. pacificus*, *G. polynesiensis*, *G. caribaeus*, *G. carolinianus*, *G. carpenteri*, *G. excentricus*, *G. scabrosus*, *G. silvae*, *G. balechii*, *G. cheloniae*, *G. lapillus*, *G. honu*, *G. jejuensis*, *G. lewesii*, and *G. holmesii* [2,3,4,5,6,7,8,9,10,11,12]. Two *Gambierdiscus* species were reclassified and placed into the genus *Fukuyoa*, i.e., *F. yasumotoi* and *F. ruetzleri* based on their morphological and phylogenetic divergence. Subsequently, two additional species, *F. paulensis* and *F. koreansis*, were also identified [13,14,15]. These microalgae are able to produce a suite of polyketide secondary metabolites, including liposoluble compounds, i.e., ciguatoxins (CTXs) [16,17,18,19,20,21,22,23,24] versus more hydrosoluble compounds, i.e., maitotoxins (MTXs) [25,26,27,28,29,30,31,32,33,34,35,36], gambieric acids [37,38], gambierol [39,40], gambieroxide [41], and gambierones [42,43,44,45,46,47,48,49,50]. While CTXs and, to a lesser extent, MTXs, have been implicated in CP [51,52,53], it is not fully understood if the other metabolites could also play a role in CP, and most of them are considered compounds of interest due to their bioactivity and/or potential therapeutic applications [54,55,56,57,58,59,60,61,62]. *Gambierdiscus*/*Fukuyoa* populations preferentially grow within turf algae covering degraded coral substrates, but sand, coral, detritus and other surfaces were also found to be potential habitats for these microalgae [51]. The CTXs produced by these microalgae enter the food chain after the grazing of *Gambierdiscus*/*Fukuyoa* cells by herbivorous fish, are transferred into carnivorous fish through predation, and eventually end up in consumers [63,64]. After eating contaminated fish, CP can cause serious digestive, cardiac and neurologic symptoms, making CP one of the most common food-borne diseases, affecting between 50,000 and 500,000 people per year worldwide [65]. During CTX transfer along the food chain, biotransformation and biomagnification processes underwent by algal CTXs lead to a variety of CTX analogs, with around 30 analogs described for the Pacific Ocean (P-CTXs), 12 for the Caribbean Sea (C-CTXs) and 6 for the Indian Ocean (I-CTXs) [51,66,67]. While the link between algal CTX production and those observed in fish is well-established in the Pacific region based on the bio-oxidation process of these toxins [68], this process still needs to be verified for the Caribbean and the Indian regions, as no C-CTXs and I-CTXs have been found in *Gambierdiscus*/*Fukuyoa* spp. from these regions to date.

To strengthen CP research around the world, the Inter-Agency Global Ciguatera Strategy for Improved Research and Management recommends focusing on all aspects of CP, including improving toxin detection in cells of *Gambierdiscus*/*Fukuyoa* species [69]. The detection of CTXs remains challenging, as they are produced from femtograms to picograms in these microalgae [67,70]. One of first methods that was developed was the in vivo mouse biological assay, used to search for the presence of CTXs in wild cells and in vitro cultures of *Gambierdiscus*/*Fukuyoa* [3,8,9,11,17,32,71,72,73,74,75,76,77,78,79]. However, since 2015, this method is no longer recommended for the detection of liposoluble toxins by the European Union. Instead, the use of alternative methods is supported [80], such as in vitro assays based on the CTXs’ mode of action, which bind to site 5 of the alpha subunit of voltage-gated sodium channels (VGSCs) [81,82,83]. As a result, the radioactive receptor binding assay (rRBA) [17,84,85,86,87,88,89] and, more widely, the neuroblastoma cell-based assay (CBA-N2a) [5,19,20,22,90,91,92,93,94,95,96,97,98,99,100,101,102] have successfully been applied to the *Gambierdiscus*/*Fukuyoa* matrix for the qualitative and quantitative detection of CTXs. These biological methods are able to detect the composite effect of all CTX analogs present in the sample, knowing that CTX-producing species such as *G. polynesiensis* are able to synthesize up to 10 CTX analogs [23]. However, characterizing the different CTX analogs and CTX profiles specific to *Gambierdiscus*/*Fukuyoa* spp. requires analytical methods such as high-performance liquid chromatography coupled with UV or fluorescence detection (HPLC-UV or HPLC-FLD), and liquid chromatography tandem mass spectrometry (LC-MS/MS) [9,16,17,18,22,23,24,45,78,79,103,104,105,106,107,108,109], since biological methods cannot discriminate between CTX analogs [110]. More recently, the development of immuno-biosensor tools has also been undertaken [111,112], allowing for two series of CTX congeners, CTX1B and CTX3C, to be discriminated. This technique has been applied to samples of *Gambierdiscus* and *Fukuyoa* according to Gaiani, et al. [113,114]. At present, the most common approach is the use of a functional biological method that measures the composite toxicity in samples, followed by an analytical technique such as LC-MS/MS to confirm the identity of CTX compounds in positive samples.

The objective of this study was to compare the performance of two functional, biological methods based on CTX’s mode of action, i.e., the fluorescent RBA (fRBA) and CBA-N2a, versus a chemical method, i.e., LC-MS/MS for the detection and quantification of CTXs in *Gambierdiscus* matrix. Since fRBA was applied for the first time to *Gambierdiscus* matrix, different protocol parameters were refined, while recently improved protocols were applied in the case of CBA-N2a [115] and LC-MS/MS analyses [21]. A total of 30 samples of *G. polynesiensis* were selected according to their CTX contents, as determined from crude extracts using LC-MS/MS [23]. Prior to analyses, samples were subjected to an extraction step using liquid/liquid partitioning for the separation of lipophilic toxins (CTXs) from hydrophilic toxins (e.g., MTXs, gambierones) [17], thus limiting the potential crossover effects of these latter compounds on functional targets [43]. In addition, to ensure accurate comparison between methods, the same standard and same *G. polynesiensis* extract were analyzed by all three methods to avoid potential biases due to the use of i) different origin/batches of standard CTXs, and ii) different chemical extraction protocols with varying efficiency and recovery percentages. Finally, quantifications of each *Gambierdiscus* sample were undertaken in three independent experiments on different days by all three methods. The mean ± standard deviation for each of three independent experiments was then calculated, and those values were statistically analyzed and compared. The strengths and weaknesses of the three methods are further discussed.

## 2. Results and Discussion

### 2.1. Binding Affinity of CTX3C and Gambierdiscus polynesiensis Extracts Using the fRBA

The RBA is based on the competition for site 5 of VGSCs between CTXs and a brevetoxin (PbTx) labelled with radioactive ([^3^H]PbTx-3) or fluorescent (Bodipy^®^-PbTx2) compounds [81,116]. When CTX concentrations increase, the binding of labelled brevetoxin to receptors decreases, which leads to a decrease in radioactivity (rRBA) or fluorescence (fRBA) signal in a dose-dependent manner. This assay measures the composite binding affinity for VGSC receptors of all CTX analogs present in a given biological sample. Since fRBA was applied for the first time to *Gambierdiscus* matrix, several parameters were evaluated before the quantification of *G. polynesiensis* toxic samples.

#### 2.1.1. Solvent Effect, Incubation Parameters, and Matrix Effects

First, the effects of three solvents commonly used to resuspend CTX standards or biological extracts were evaluated. Our findings indicated that these three solvents, ethanol (EtOH), methanol (MeOH), or dimethylsulfoxide (DMSO), can be used by up to 40% without inducing any unspecific effect (Figure S1).

**Figure 1:**
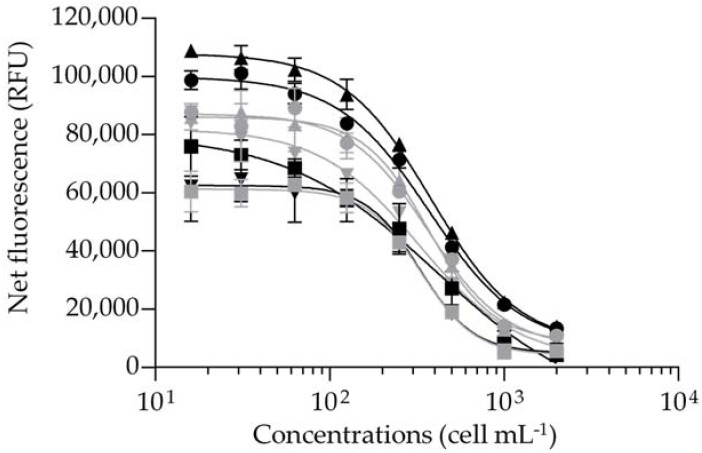
Binding dose–response curves of *Gambierdiscus polynesiensis* TB92 sample extracts using the fluorescent receptor binding assay with an incubation step at 37 °C for 30 min (grey) versus 4 °C for 90 min (black). Data represent the mean ± standard deviation (SD) (each concentration run in duplicate wells) of four independent experiments run on different days. The mean ± SD of EC_50_ values obtained for TB92 were 349 ± 21 and 359 ± 26 cell mL^−1^ (*n* = 4) with coefficients of variation (CV) of 6% and 7% incubated at 37 °C for 30 min and 4 °C for 90 min, respectively.

Second, the temperature and duration of the fRBA incubation step were refined by incubating *G. polynesiensis* TB92 sample extract at 4 °C for 90 min versus 37 °C for 30 min (Figure 1).

The effective concentration at 50% (EC_50_, defined as the concentration of competitor at which the binding of the fluorescently labelled PbTx-2 is reduced by half) obtained under the two incubation conditions did not show any significant difference between the two tested temperatures and incubation durations. In most RBA-based studies, the binding reaction usually occurs at a temperature of 4 °C from 60 to 120 min for the detection of both VGSC activators, i.e., CTXs and PbTxs [17,81,83,84,85,87,89,117,118,119,120,121,122,123,124,125,126,127], and VGSC inhibitors, i.e., saxitoxins [128,129,130,131,132,133], but also gambierol and gambieric acid-A [54], and brevenal [134,135]. Our findings indicated that an incubation at 37 °C for 30 min showed the same efficacy in the detection and quantification of CTXs in *G. polynesiensis*, so this protocol was further applied in the following experiments. Of note, McCall, et al. [116] have shown that binding equilibrium was reached after approximately 30–40 min at 4 °C in fRBA experiments.

Third, to evaluate the maximum concentration of a matrix that does not induce unspecific effects (MCE), four strains that were previously found to be negative by CBA-N2a (data not shown) were extracted using the same chemical protocol [17] and tested at eight serially diluted (1:2) concentrations. During binding experiments, a slight decrease in fluorescence began at 32,000 cell mL^−1^ for *G. toxicus* RIK31 and TKU5 strains, while no decrease was observed for *G. toxicus* HPT3 and *G. pacificus* TKR9 at up to 64,000 and 128,000 cell mL^−1^. To avoid any false-positive binding, the MCE was set at 16,000 cell mL^−1^ (Figure S2). This value is higher than the highest concentration of 7500 cell mL^−1^ tested for *Gambierdiscus* extract using the rRBA in two previous studies [87,88].

#### 2.1.2. Efficiency of the fRBA When Applied to CTX3C and *Gambierdiscus polynesiensis* Samples

**Figure 2:**
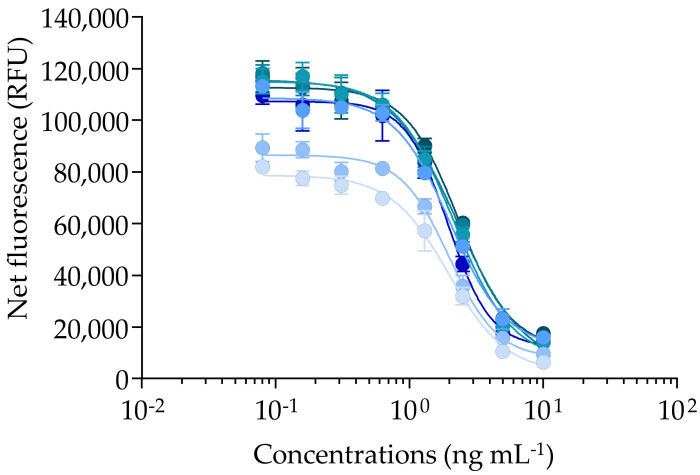
Binding dose–response curves of CTX3C using the fluorescent receptor binding assay (fRBA). Data represent the mean ± standard deviation (SD) (each concentration run in triplicate wells) of seven independent experiments run on different days (incubation 30 min at 37 °C, synaptosomes at 0.1 mg mL^−1^, and Bodipy PbTx2 at 1 nM). The mean ± SD of EC_50_ value for CTX3C was 2.10 ± 0.16 ng mL^−1^ (*n* = 7) with coefficient of variation (CV) of 7.6%.

The Pacific ciguatoxin CTX3C showed a response typical of the mode of action of VGSC activators, illustrated by a sigmoidal dose–response curve with a negative slope (Figure 2).

The full competitive binding curves of CTX3C allowed us to estimate the EC_80_, EC_50_, and EC_20_ values (*n* = 7) with coefficients of variation (CV) ranging from 7.6 to 14.5 % with good repeatability (Table 1), although RFU values did vary from one experiment to another (Figure 2). As per Van Dolah, et al. [131], the CV or relative standard deviation (RSD) of toxin standards data from three independent experiments should remain below 30%. In our conditions, the mean EC_50_ value (LOQ) of CTX3C was three-fold higher than those previously reported using the fRBA [136] or the rRBA [84,91], even though the EC_20_ and EC_80_ were comparable to several previous studies (Table 1).

Such differences could be explained by the use of different batches of CTX3C or standards of distinct origins in previous studies, as well as different concentrations of synaptosomes and labelled PbTx (fluorescent Bodipy^®^-PbTx2 or radioactive [^3^H]PbTx-3). However, despite the different origins of CTX3C (ILM versus Wako), incubation conditions, and labelling of PbTx-2 or PbTx-3, our results were consistent with rRBA results that were recently published by Díaz-Asencio, et al. [126], showing similar EC_80_, EC_50_, and EC_20_ values of CTX3C (after their conversion into ng ml^−1^ unit) (Table 1). In our conditions, the limits of detection and quantification of the fRBA applied to the *Gambierdiscus* matrix were LOD = 68.53 ± 5.25 and LOQ= 131.32 ± 9.94 fg CTX3C eq cell^−1^ (Table 1), which are within the range of values previously reported for the rRBA [84,87,88,126].

**Figure 3:**
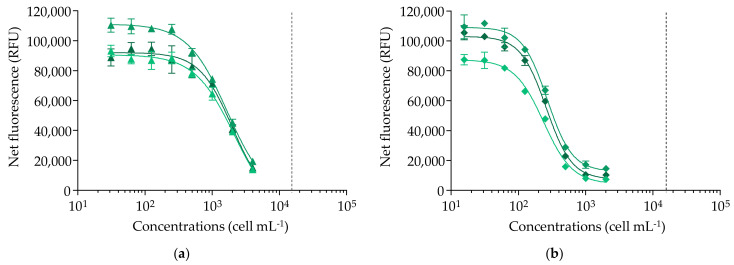
Binding dose–response curves of *Gambierdiscus polynesiensis* using the fluorescent receptor binding assay (fRBA). (**a**) RG92-b sample (▲). (**b**) NHA4-b sample (◆). Data represent the mean ± standard deviation (SD) (each concentration run in triplicate wells) of three independent experiments run on different days. The dotted vertical line corresponds to the maximum concentration of cell equivalent (MCE = 16,000 cell mL^−1^) for matrix interference. The mean ± SD of EC_50_ values obtained for RG92-b and NHA4-b were 1926 ± 237 and 255 ± 16 cell mL^−1^ (*n* = 3) with coefficients of variation (CV) of 14% and 8%, respectively (incubation for 30 min at 37 °C).

When applied to the 30 samples of *G. polynesiensis*, competitive binding curves with a negative slope were obtained below the MCE from the least toxic to the most toxic sample, i.e., RG92-b and NHA4-b, respectively (Figure 3).

The EC_50_ values of the 30 *G. polynesiensis* samples ranged from 255 ± 16 to 1926 ± 237 cell mL^−1^, with CV varying from 3 to 14%, except for two outliers (Table 2). The CTX concentrations estimated in the 30 samples varied by 7.5-fold, from the lowest to the highest value, i.e., 1.10 ± 0.15 to 8.27 ± 0.68 pg CTX3C eq cell^−1^, respectively, with CV ranging from 2 to 18% showing good repeatability, except for the same two outliers (Table 2). With most CVs < 15%, these fRBA data for *G. polynesiensis* samples can thus be considered reliable, except for two samples, RG92-a and RAI1-f, which showed CVs of 51 and 69% and 49 and 38% for EC_50_ values and toxin content, respectively (Table 2). These observations warrant additional analyses to explain such discrepancies.

When considering the mean CTX content of each strain, RG92 (*n* = 3), RIK7 (*n* = 12), RAI1 (*n* = 8), and NHA4 (*n* = 7), regardless of the ages of the cultures and culture conditions (Tables S1 and S2), significant differences were observed between strains, except between RIK7 and RAI1 samples. By way of example, the mean CTX content of RG92, i.e., 1.60 ± 0.82 pg CTX3C eq cell^−1^ was the lowest, followed by RIK7 and RAI1, which were similar, 4.06 ± 1.36 versus 5.13 ± 1.03 pg CTX3C eq cell^−1^, while NHA4 showed the highest CTX content of 7.90 ± 0.51 pg CTX3C eq cell^−1^. Of note, ranges of from 1.10 ± 0.15 to 2.04 ± 1.40 and 3.63 ± 0.45 to 5.87 ± 0.73 pg CTX3C eq cell^−1^ for the three RG92 samples and the eight RAI1 samples, respectively, were consistent with the CTX contents of 2.8 and 4.4 pg CTX3C eq cell^−1^ estimated by rRBA for *G. polynesiensis* RG92 and RAI1, respectively, cultured in standard conditions as defined in Chinain, et al. [17].

### 2.2. Cytotoxic Activity of CTX3C and Gambierdiscus polynesiensis on N2a cells Using the CBA-N2a

**Figure 4:**
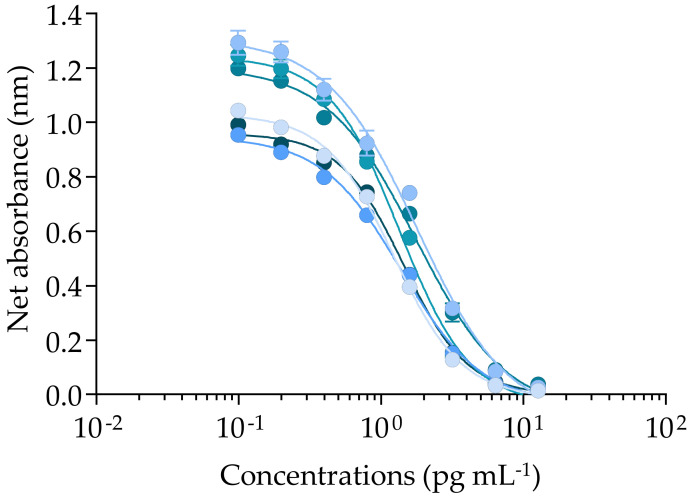
Cytotoxic dose–response curves of CTX3C under OV+ condition (100/10 µM) using the neuroblastoma cell-based assay (CBA-N2a). Data represent the mean ± standard deviation (SD) (each concentration run in triplicate wells) of six independent experiments run on different days. The mean ± SD of EC_50_ value for CTX3C was 1.50 ± 0.23 pg mL^−1^ (*n* = 6), with coefficient of variation (CV) of 15.6%.

As CTXs have no direct cytotoxic effect on neuroblastoma (N2a) cells, their detection requires the addition of the sodium/potassium (Na^+^/K^+^) ATPase pump blocker ouabain (O), together with the sodium-channel activator veratridine (V), which induces permanent activation of the VGSCs without cytotoxicity (non-destructive OV treatment) [115]. When CTX concentrations increase, a progressive decrease in N2a cell viability is observed in the presence of OV treatment (OV+ condition), whereas no cytotoxic effect is detected in the absence of OV treatment (OV− condition) [115]. This specific mode of action of VGSC activators results in a sigmoidal dose–response curve with a negative slope in OV+ condition, as illustrated by the curve of Pacific CTX3C (Figure 4). Conversely, N2a viability is maintained in OV− conditions regardless of CTX concentration (data not shown) [115]. The EC_80_ and EC_50_ values of CTX3C were estimated at 0.63 ± 0.05 and 1.5 ± 0.23 pg mL^−1^ (*n* = 6) with CVs of 8.2 and 15.6%, respectively, showing overall good repeatability (Figure 4), as in Viallon, et al. [115].

These EC_80_ and EC_50_ values were consistent with those previously reported for CTX3C in some studies [89,91,96,109,115,136], but differed by up to three-fold with other studies (Table 3) [19,137,138,139]. Again, such differences could be attributed to the use of different batches of CTX3C or standards of distinct origins, as well as the striking diversity of CBA-N2a protocols that are actually available in the literature, which makes comparisons between studies difficult [115]. The LOD of CBA-N2a for CTX3C was three-fold higher than the one found by Litaker, et al. [94] and the LOD and LOQ values in the *Gambierdiscus* matrix were two-fold higher than previous studies (Table 3) [22,96,140].

**Figure 5:**
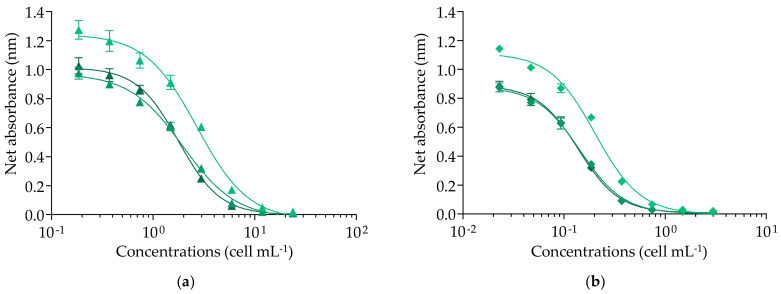
Cytotoxic dose–response curves of *Gambierdiscus polynesiensis* in OV+ conditions (100/10 µM) using the neuroblastoma cell-based assay (CBA-N2a). (**a**) RG92-b sample (▲). (**b**) NHA4-g sample (◆). Data represent the mean ± standard deviation (SD) (each concentration run in triplicate wells) of three independent experiments run on different days. The maximum concentration of cell equivalent (MCE = 1904 cell mL^−1^) for matrix interference was not indicated in both graphs, being outside the concentration range tested for these samples. The mean ± SD of EC_50_ values for RG92-b and NHA4-g were 2.08 ± 0.46 and 0.15 ± 0.04 cell mL^−1^ (*n* = 3) with coefficients of variation (CV) of 4 and 17%, respectively.

The CBA-N2a measures the composite cytotoxicity induced on N2a cell viability by all CTX analogs present in a given biological sample. As observed for CTX3C, sigmoidal dose–response curves with a negative slope in OV+ conditions were obtained (below the MCE) for all 30 samples, from the lowest RG92-a and RG92-b to the highest NHA4-g toxic samples (Figure 5). In this study, the samples were not tested in OV− conditions, since strains of *G. polynesiensis* such as NHA4 showed a typical response similar to that of CTX3C in CBA-N2a under OV− and OV+ conditions [97,115].

The EC_50_ values ranged from 0.15 ± 0.04 to 2.28 ± 0.54 cell mL^−1^, with CV varying from 12 to 30%, except for the NHA4-d sample, for which a CV of 38% was observed (Table 4). The CTX concentrations estimated in the 30 samples varied by 14.2-fold from the lowest to the highest value, i.e., 0.68 ± 0.03 to 9.72 ± 1.64 pg CTX3C eq cell^−1^, with CV ranging from 4 to 27%, showing good repeatability (Table 4).

When considering the mean CTX content in each *G. polynesiensis* strain, RG92 (*n* = 3), RIK7 (*n* = 12), RAI1 (*n* = 8), and NHA4 (*n* = 7), regardless of culture ages and culturing conditions (Tables S1 and S2), significant differences were observed across strains, except between RG92 and RIK7, and RIK7 and RAI1. More precisely, the lowest CTX content (mean value) was that of RG92 (0.71 ± 0.08 pg CTX3C eq cell^−1^), followed by those of RIK7 and RAI1, i.e., 2.29 ± 0.86 versus 2.96 ± 0.83 pg CTX3C eq cell^−1^, with NHA4 displaying the highest mean value of 5.69 ± 2.08 pg CTX3C eq cell^−1^ (calculated from Table 4). Our findings are in agreement with those of Longo et al. [22], showing that RG92 was significantly less toxic than RIK7, RAI1, and NHA4 when these strains were maintained in standard culture conditions, an observation likely linked to the higher age of this strain (Table S1) [22].

Overall, the CTX contents of all these 30 samples of *G. polynesiensis* were consistent with the range of 1.10–20.9 pg CTX3C eq cell^−1^ reported in this species in previous CBA-N2a studies [22,23,32,91,96,97].

### 2.3. Detection and Quantification of CTX3C and Gambierdiscus polynesiensis Using LC-MS/MS

**Figure 6:**
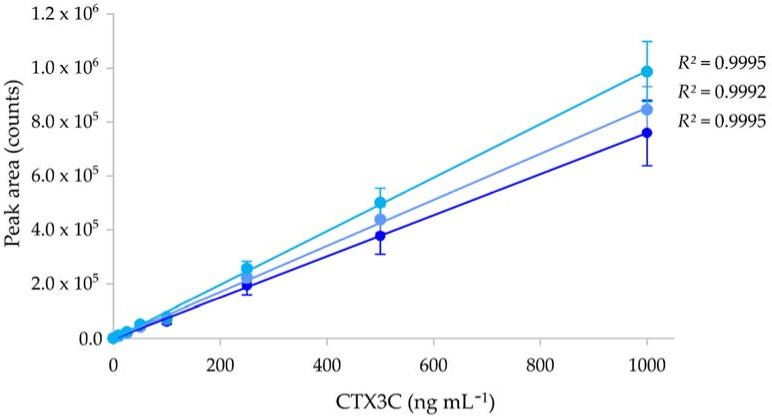
Calibration curves of CTX3C using liquid chromatography tandem mass spectrometry (LC-MS/MS). Data represent the mean ± standard deviation (SD) (each concentration run in triplicate) of three independent experiments run on different days (*n* = 3).

The LC-MS/MS is a chemical method for detecting, identifying and quantifying molecules of interest by measuring their mass-to-charge ratio (*m*/*z*) and analyzing their fragmentation pathways. The Multiple Reaction Monitoring (MRM) used in this study is a sensitive and specific technique that can selectively quantify compounds within a complex matrix. Its principle resides in (i) the selection of a charged ion corresponding to the molecule of interest (parent or precursor ion), (ii) the fragmentation of this parent ion to produce a range of daughter ions, and (iii) the selection of one or more of these daughter ions for quantification. Using LC-MS/MS, the LOD and LOQ values for CTX3C were determined with the ordinary least-squares regression data method [141] at 2 and 6 ng mL^−1^ (*n* = 3) according to method 2, as described in Sibat, et al. [21] (Figure 6).

The LC-MS/MS calibration curves for CTX3C showed a good correlation, with *r*^2^ varying between 0.9992 and 0.9995 (Figure 6). Although very few LC-MS/MS studies found in the literature actually provide LOD and LOQ values for CTX standards, the LOD and LOQ values found in the present study are in the range of the values reported in previous studies (Table 5).

**Figure 7:**
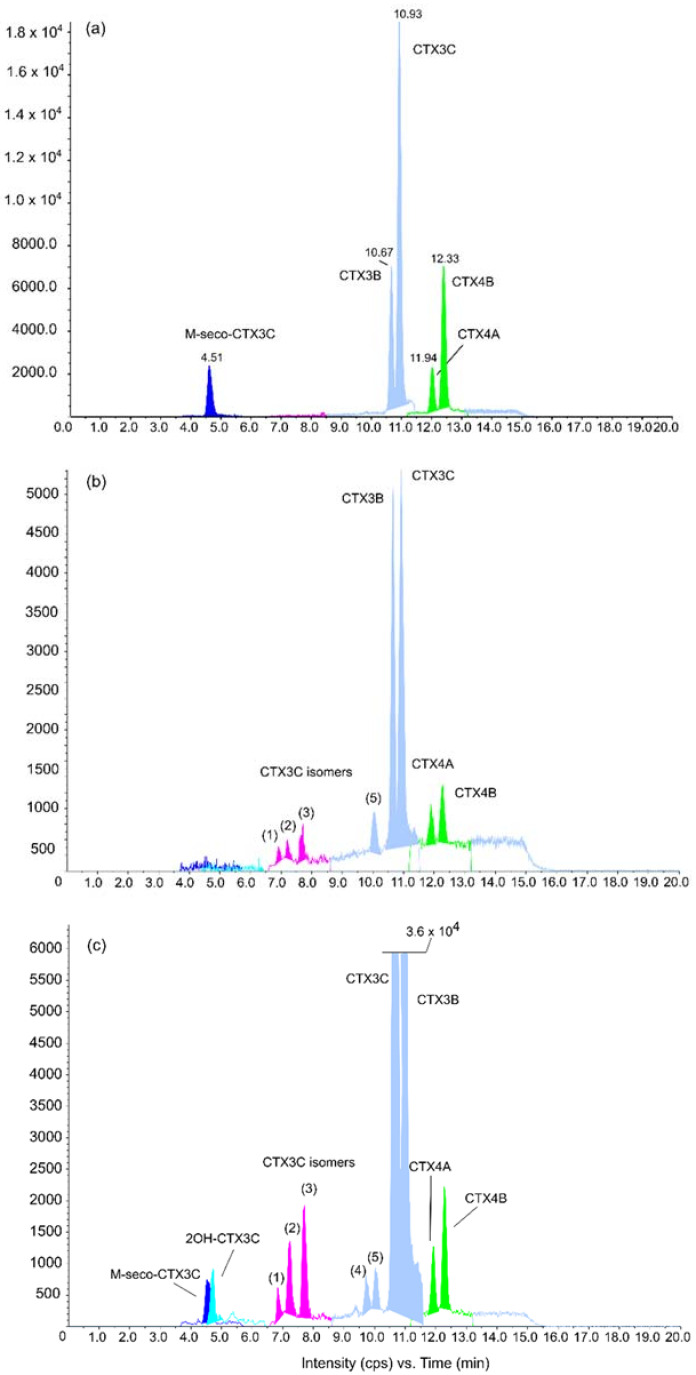
LC-MS/MS chromatograms of ciguatoxin (CTX) standards and *Gambierdiscus polynesiensis* using the most intense MRM transition for each toxin. (**a**) Mix of CTX standards; (**b**) RG92-b sample; (**c**) NHA4-d sample. Data represent toxin profiles from three independent experiments run on different days (*n* = 3). As peaks (1) to (5) share the same MRM transitions as CTX3C (Table S3), they are identified as CTX3C isomers.

The LC-MS/MS measures the abundance of a compound (here a CTX analog) that has been previously separated by liquid chromatography in a sample. The ionic abundance is represented by a chromatographic peak with a retention time and a peak area (Figure 7). Regarding the diversity of algal Pacific CTXs present in the 30 samples, from five to 12 algal CTX analogs were detected in the least toxic (RG92-b) vs. most toxic (NHA4-f) samples, respectively, as shown in Figure 7. More specifically, CTX3B, CTX3C, CTX4A, and CTX4B were detected in all sample extracts while eight additional analogs were present only in certain samples, i.e., from the most to the least frequently detected analog: analog 3 > analog 2 > analog 5 > analog 4 > 2OH-CTX3C > analog 1> M-seco-CTX3C > 3OH-CTX3C (Figure 7 and Table 6).

The CTX profile of all *G. polynesiensis* samples was mainly composed of CTX3B and CTX3C, ranging from 56 to 91% of the total CTX content, with CTX3B as the dominant congener compared to CTX3C, as previously highlighted by Longo et al. [22]. Nonetheless, CTX3C was the dominant algal CTX in two samples, RG92-b and RG92-c (Table 6).

The CTX contents of the 30 samples varied by 8.17-fold from the lowest to the highest value, i.e., from 1.01 ± 0.14 to 8.25 ± 0.45 pg CTX3C eq cell^−1^ with CV ranging from 0% to 14%, showing good repeatability (Table 6). When considering the mean CTX content in each strain, regardless of the age of cultures and culture conditions (Tables S1 and S2), significant differences were observed across strains, except between RIK7 and RAI1. The lowest CTX content (mean value) was observed in RG92 (1.09 ± 0.13 pg CTX3C eq cell^−1^), followed by RIK7 (3.67 ± 1.38 pg CTX3C eq cell^−1^) and RAI1 (4.17 ± 1.08 pg CTX3C eq cell^−1^), with NHA4 strain displaying the highest concentration (7.04 ± 0.53 pg CTX3C eq cell^−1^). These results confirmed the hierarchy relative to CTX-production among these strains, as previously outlined by Longo et al. [22,23]. Nevertheless, the CTX contents obtained in the 30 samples extracted using the liquid/liquid partition are higher than those obtained from the crude extracts of the same samples of RG92, RIK7, RAI1 and NHA4 in the study of Longo, et al. [23], probably due to the use of different extraction protocol procedures.

With CTX levels ranging from 0.68 ± 0.03 to 9.72 ± 1.64 pg per cell estimated by the three methods, our results confirm *G. polynesiensis* as the most toxic *Gambierdiscus* species described to date, compared to toxicity levels in the range of femtograms reported in other *Gambierdiscus* species [51]. Our data also highlight the existence of an intra-specific variation in CTX production in the species *G. polynesiensis*, as also evidenced in other *Gambierdiscus* species [51].

### 2.4. Comparison of the Performance of the Three Detection Methods

Regarding the sensitivity of the three methods used, based on the LOD and LOQ values for CTX3C, the fRBA appeared to be from 1.8 to 2.9 times more sensitive than LC-MS/MS (Table 1 and Table 5). Conversely, the CBA-N2a was from 1746 to 1400 and 3175 to 4000 times more sensitive than fRBA and LC-MS/MS, respectively (Table 1, Table 3 and Table 5). The opposite results were observed for the detection of PbTx standards, with the rRBA being more sensitive than CBA-N2a [122]. Depending on the CTX analogs and methods used, the CBA-N2a (LOD = 0.63 ± 0.05 pg mL^−1^) was also more sensitive than the colorimetric ELISA (LOD value of CTX3C = 2 pg mL^−1^), the immunosensor and immunoassay (LOD = 1.96 and 3.59 versus 3.29 and 6.17 pg mL^−1^ for CTX1B and 51-OH-CTX3C, respectively) [111,112].

When comparing the sensitivity for CTX detection in *Gambierdiscus* matrix, the difference between the three methods was less important than for the pure toxin CTX3C (Table 1, Table 3 and Table 5). Regarding LOD and LOQ values, the fRBA appeared to be from 3.4 to 2.2 times less sensitive than LC-MS/MS (Table 1 and Table 5), while the CBA-N2a was from 61 to 76 and 208 to 166 times more sensitive than LC-MS/MS and fRBA, respectively (Table 1, Table 3 and Table 5). Conversely, LC-MS/MS proved to be more sensitive than CBA-N2a and rRBA for the detection of PbTxs in the dinoflagellate *Karenia brevis* [142]. Our results are also in agreement with previous studies showing that LC-MS/MS and/or CBA-N2a were more sensitive than rRBA or fRBA for the detection of CTXs in fish [87,89,127,136,143].

In addition, our results show that LC-MS/MS presents the best repeatability, with an average CV of 6.0 ± 3.4% against 12.1 ± 12.7% and 13.6 ± 5.6% for fRBA and CBA-N2a, respectively (not considering the two extreme CV values >30% for fRBA). These differences in repeatability could be explained, in part, by the fundamental differences related to the principles of these three methods (chemical detection methods vs. functional, biological methods).

**Figure 8:**
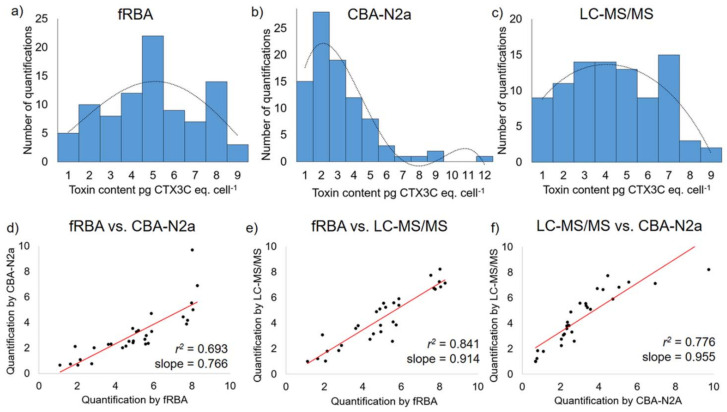
Comparison of CTX contents in 30 *Gambierdiscus polynesiensis* samples; each sample tested by the fluorescent receptor binding assay (fRBA), neuroblastoma cell-based assay (CBA-N2a), and liquid chromatography tandem mass spectrometry (LC-MS/MS) in three independent experiments (*n* = 3) for each method. The distribution of CTX contents measured by the three methods is presented in (**a**–**c**), the scatter plots and regression lines of CTX contents between two methods in (**d**–**f**), with corresponding correlation coefficients (*r*^2^) and slopes listed in each graph.

When comparing mean CTX contents for the 30 samples of *G. polynesiensis* between the three methods, there was a significant difference among the three assays (*p* < 0.05), although variations in toxicity among samples led to similar means when grouped together across assays (Table 7). The fRBA and LC-MS/MS showed a similar range of CTX contents (minimum–maximum), while CBA-N2a showed a wider range of values, yielding the lowest and the highest CTX contents (Table 7 and Figure 8).

Figure 8 presents the distribution of CTX contents in 30 *G. polynesiensis* samples measured by each of the three methods, fRBA, CBA-N2a, and LC-MS/MS, in three independent experiments (*n* = 3), generating *n* = 90 values of CTX contents. The distribution of all CTX contents obtained with fRBA and LC-MS/MS were similar and differed from that obtained with CBA-N2a (Figure 8). When methods were compared two by two, all three assays were significantly correlated with each other, with the highest correlation coefficient (*r*^2^ = 0.841) found between the fRBA and LC-MS/MS (Figure 8).

Similar findings were observed in the detection of paralytic shellfish toxins such as saxitoxins in dinoflagellates and shellfish extracts using these three methodological approaches, with a significant correlation found between rRBA and HPLC, and between rRBA and LC-MS [132,133].

When comparing the CTX contents of each strain between the three methods, no significant differences were observed for RG92 (*p* value = 0.0997), but this is likely due to the limited sample size (*n* = 3 samples). Conversely, significant differences in toxicity estimates were observed between CBA-N2a and fRBA for RIK7, RAI1, and NHA4, while no difference was observed between fRBA and LC-MS/MS for any of the three strains. The CBA-N2a also yielded significant differences in toxicity estimates compared to LC-MS/MS for strains RIK7 and RAI1, as was also found by Longo, et al. [22]. In this study, despite the use of the same batch of CTX3C standard and the same chemical extract of each sample tested by the three methods, differences were still observed. These findings are of importance, as a lot of studies report differences in discrete numerical values of toxicity estimates [22,133,144]. The most likely explanation for these observations is that comparisons must be relative: the different assays measure different parameters, making cross-comparisons of precise numerical concentrations difficult. All assays must be normalized to what regulatory agencies define as “toxic.” However, it is important to note that relative estimates of CTX contents were the same in this study, as demonstrated by the good correlations observed (i.e., high *r*^2^ values). In other words, high toxicity samples measure high on all assays, while low toxicity samples measure low (Figure 8).

A preliminary literature review reveals that very few studies exploring the correlations between CTX quantification methods in *Gambierdiscus* are actually available, and that most studies focus on the comparison of CTX quantifications in fish. Concerning the correlation between both functional methods, our results are in agreement with previous studies showing a significant correlation between rRBA/fRBA and CBA-N2a for the detection of C-CTXs in fish with *r*^2^ ≈ 0.7 [122,136,143]. In those studies, rRBA/fRBA yielded higher CTX equivalents on average, suggesting that fish samples may contain multiple CTX congeners with varying affinities to sodium channels. Consequently, equivalent amounts of bound toxins from the various samples may produce varying degrees of cytotoxicity [136,145]. Similar observations were reported for the detection of P-CTXs in fish from Rapa Island [89]. However, a recent study showed contradictory results, with CBA-N2a giving higher P-CTX equivalents than rRBA in three deep-water fish specimens, while no significant difference was observed between rRBA and CBA-N2a estimates for one specimen [127]. The CBA-N2a also showed a significant correlation with two immunosensing tools for the detection of P-CTXs in fish [112]. However, these results should be interpreted with caution, as different chemical extraction protocols were applied depending on the detection method used. When comparing functional versus chemical methods, a strong correlation was observed between CBA-N2a and LC-MS/MS quantification of carnivorous fish [146], while, in two other studies, no correlation was observed between the quantifications of CBA-N2a and LC-MS/MS in carnivorous fish, with LC-MS/MS giving lower estimates due to the absence of available CTX standards [127,147].

### 2.5. Practical Considerations for Algal-Based Ciguatera Risk Monitoring Programs

Our results showed the great sensitivity and repeatability of fRBA, CBA-N2a and LC-MS/MS when applied to the detection of CTXs in *Gambierdiscus* samples. With a detection threshold of around 0.3 fg of CTX per cell, the CBA-N2a appears to be the best screening method for *Gambierdiscus* species, especially those producing very low amounts of CTXs. Two-hundred times less sensitive, but also more specific and less time-consuming (2 h for fRBA assay versus 2 days for CBA-N2a), fRBA can detect moderate to high CTX levels (≥100 fg CTX eq cell^−1^) and is considered as a complementary detection method to CBA-N2a. However, its high requirements regarding the amount of standard material, i.e., 50 ng per experiment for fRBA versus 60 pg and 29 ng for CBA-N2a and LC-MS/MS, respectively, potentially limits the use of fRBA to laboratories with large amounts of CTX standards, as is already the case with rRBA. Similarly, for the lowest toxic *G. polynesiensis* sample, the highest cell biomass required for one experiment was 20,000 cells by fRBA, versus 100 and 500 cells for CBA-N2a and LC-MS/MS, respectively. The great advantage of both in vitro methods is their high-throughput format allowing for (i) the qualitative screening (presence or absence of composite CTX-like activity) of 120 samples at a time, tested at a single concentration), and (ii) the quantification of CTX contents in 15 positive samples at a time, if tested at a range of eight distinct concentrations.

In algal-based ciguatera risk-monitoring programs, an efficient approach would be to carry out a qualitative screening using CBA-N2a and/or fRBA to quickly discriminate between negative samples and those showing from low to high ciguatoxicity. This approach allows for a rationalization in testing efforts by focusing only on CTX-positive samples in quantitative analyses. With an intermediate analysis time of 23 h for accurate quantification and only 20 min for qualitative analysis, LC-MS/MS is still more specific than fRBA and CBA-N2a because it is able to identify all known analogs of CTXs. However, its main limitation is that it is not able to detect unknown CTX analogs, whereas the composite activity of both known and unknown CTX analogs can be detected by fRBA and CBA-N2a.

## 3. Materials and Methods

### 3.1. Gambierdiscus Isolates

This study used several strains of *G. polynesiensis* (TB92, RG92, RIK7, RAI1, and NHA4), *G. toxicus* (HPT3, RIK31, and TKU5) and *G. pacificus* (TKR9) originating from different islands and/or archipelagos of French Polynesia (Table S1). These strains were isolated from macro-algal and/or artificial substrate (window screen) samples, collected following the methods described in Chinain, et al. [17] and Tester, et al. [148], respectively. All strains are part of the Laboratory of Marine Biotoxins culture collection at the Institut Louis Malardé (ILM, Tahiti, French Polynesia), where cultures are deposited.

### 3.2. Reagents and Chemicals

Chemicals used for extraction, toxins and samples resuspension and dilution were methanol (MeOH), ethanol (EtOH), and dichloromethane (CH_2_Cl_2_) (AnalaR Normapur^©^ACS, VWR) and dimethylsulfoxide (DMSO) HPLC grade (PanReac Applichem, VWR).

Ciguatoxin CTX3C sourced from the Institut Louis Malardé (ILM) bank of CTX standards and was obtained from *G. polynesiensis* TB92 strain following purification by high-pressure liquid chromatography (HPLC), as detailed in Chinain, et al. [17]. This toxin was dissolved in MeOH or EtOH to produce a stock solution at 1 µg mL^−1^, 20 ng mL^−1^, and 2 µg mL^−1^ for fRBA, CBA-N2a and LC-MS/MS, respectively. All toxins were stored at −20 °C. Gilson Microman positive-displacement pipettes (Gilson, Inc., Middleton, WI, USA) were used to transfer all the solvent solutions used in the assay for greater accuracy.

### 3.3. In Vitro Cultures

Samples from TB92 *G. polynesiensis*, TKR9 *G. pacificus* and HPT3, RIK31, and TKU5 *G. toxicus* strains were obtained under routine culturing conditions, as described in [17]. As for *G. polynesiensis* strains (RIK7, NHA4, RAI1, and RG92), samples were cultured under six different conditions, three conditions using nitrate as N source at three distinct pH values (7.9, 8.2, and 8.4), and the three others using urea (Sigma-Aldrich, St. Louis, MO, USA) as described in Longo et al. [23]. Samples selected for this study corresponded to distinct N:P ratios (i.e., 24 and 48) and three culture ages, i.e., late exponential, early stationary, and senescence growth phases, and were selected based on two main criteria: (i) they represented cell biomasses ranging from ≈ 80,000 to 174,000 cells and (ii) their crude extracts contained CTX concentrations ranging from 0.47 to 4.23 pg CTX3C eq cell^−1^, as assessed by LC-MS/MS (Table S2).

### 3.4. Cells Harvest and Toxin Extraction

Prior to cell harvest, the total cell yield was determined by automated counting using a Multisizer IIITM particle-counter (Beckman Coulter, Inc., Brea, CA, USA). As described in Longo, et al. [23], cultures were harvested by filtration onto a 40-µm sieve in late exponential, early stationary, and senescence growth phases (i.e., at D10, D21, and D30 post-inoculation, respectively). Each filter was then transferred into a 50-mL Greiner tube using seawater, and centrifuged at 2800× *g* for 2 min. After discarding the supernatants, filters were freeze-dried for 20 h at −20 °C, 1 mbar, then 4 h at −60 °C, 0.01 mbar (Martin Christ, Beta 1–8 LDplus, Osterode am Harz, Germany). Each sample was then stored at −20 °C until further extraction for toxicity screening.

*Gambierdiscus* cells were extracted by adding 20 mL of MeOH directly into the tubes containing each freeze-dried cell sample. Subsequently, tubes were vortexed for 1 min and then placed in an ultrasound bath for 1 h 30. Following a centrifugation step at 2800× *g* for 10 min, the resulting supernatant was recovered in a 250 mL flask. This extraction step was repeated once in MeOH and once in aqueous methanol (MeOH:H_2_O 50/50). The three supernatants were further pooled and dried under vacuum using a rotary evaporator (Rotavapor RII, Büchi, Roubaix, France), and the resulting crude cellular extract was resuspended in 4 mL of pure MeOH. Half of this solution (2 mL MeOH) was further partitioned between 6 mL of CH_2_Cl_2_ and 2 × 3 mL of aqueous MeOH (MeOH: H_2_O 60/40) according to the procedure previously described in Chinain, et al. [17], to separate lipid-soluble compounds (e.g., CTXs) from more water-soluble compounds (e.g., maitotoxins, gambierones, etc.). The resulting dichloromethane soluble fractions (DSFs) supposed to contain CTXs were dried under vacuum, weighed, and stored at 4 °C until tested for their toxin production. The cell biomass is indicated in Table S1, and aliquots of 2000 and 20,000 cell equivalents were dedicated to CBA-N2a and LC-MS/MS, respectively, and the remaining amount to fRBA. The stock solutions of these aliquots were resuspended at a concentration of 400,000 cell mL^−1^ for fRBA, 10,000 cell mL^−1^ for CBA-N2a, and 100,000 cell mL^−1^ for LC-MS/MS.

### 3.5. Fluorescent Receptor Binding Assay (fRBA)

The fluorescent receptor binding assay (fRBA) developed in this study was based on previously published protocols [116,136,143,149], with some modifications.

#### 3.5.1. Reagents

The fRBA assay buffer was composed of 50 mM HEPES (Sigma #3375, St Louis, MO, USA), 130 mM choline chloride (Sigma C1879, St Louis, MO, USA), 5.4 mM potassium chloride (KCl, Sigma P3911, St Louis, MO, USA), 1.7 mM magnesium sulfate (MgSO4, Sigma M7506, St Louis, MO, USA), 5.5 mM glucose (Sigma G5767, St Louis, MO, USA), 6.1 mM ethylene glycol (Sigma E4378, St Louis, MO, USA). For 1 L of assay buffer, 200 μL of protease inhibitor cocktail was added (Sigma P-8340, St Louis, MO, USA). Then, the buffer was brought to pH 7.4 with Trizma base (Sigma T8524, St Louis, MO, USA) and stored at 4 °C. For each experiment, a complete assay buffer was made containing 100 mg of serum bovine albumin (Sigma B4287, St Louis, MO, USA) and one drop of Tween-20 detergent (~0.02%, Sigma P7949, St Louis, MO, USA) added to 100 mL, and mixed at room temperature for 15–20 min. The fluorescent brevetoxin (BODIPY^®^-PbTx-2) was provided by the University of North Carolina at Wilmington (UNCW) [116] and dissolved in EtOH to produce a stock solution at 0.1 mM (0.12 mg mL^−1^). Stock and intermediate (dissolved in assay buffer) solutions were covered with aluminum foil to protect from light. Synaptosomes were prepared following the protocol described by Dechraoui, et al. [83] and Darius, et al. [84] from frozen rat brains purchased at Charles River Laboratories (Les Oncins, St Germain Nuelles, France). The determination of protein content was carried out using the Bradford protein assay (Sigma B6916, St Louis, MO, USA) with bovine serum albumin (BSA) as a standard (Sigma P8119, St Louis, MO, USA) [84]. Then, the synaptosomes were resuspended in assay buffer at a stock solution of ≈11 g mL^−1^ of proteins and stored at −80 °C for use in subsequent assays.

#### 3.5.2. The fRBA Reaction

First, a Bodipy^®^-PbTx-2 mixture was prepared containing 230 µL of complete assay buffer and 50 µL of 10 nM Bodipy^®^-PbTx-2 per well and a synaptosome mixture containing 150 µL of complete assay buffer and 50 µL of 1 mg mL^−1^ of synaptosomes per well. These two mixtures were stored at 4 °C and protected from light, particularly for the mix containing the Bodipy fluorochrome. An eight-point serial 1:2 dilution of CTX3C and each *Gambierdiscus* dry extract were prepared (v = 100 µL per concentration) using a U-bottom 96-well microtiter and 20 µL of each concentration of pure toxin or *Gambierdiscus* extract were directly added in wells. All reagents were added to 96-deepwell microplates (Screen Mates, Matrix, 1 mL blocks polystyrene 4211) in the following order: 280 µL of Bodipy^®^-PbTx-2 mix (1 nM final concentration), 20 µL of CTX3C or *Gambierdiscus* extracts and 200 µL of synaptosomes mix (≈0.1 mg mL^−1^ final protein concentration) to reach a final volume of 500 µL. To avoid early binding of one of the competitors towards the sodium channel receptors, synaptosomes were added at the end and all reagents were homogenized using multichannel pipettes. Then, the 96-deepwell microplates were sealed with 96-well plate film and incubated in the dark. Before filtration, 250 µL of complete assay buffer was added to each well of a Pall Acroprep™ Advance 350 1 µm glass fiber plates (96 well format) previously placed on a Pall^®^ multi-well plate manifold vacuum apparatus and then aspirated through the filter under vacuum. After a drying step [136], 2 × 240 µL of reaction mixtures were transferred into the inner wells of the filter plates and filtered. For consistent pipetting, toxins and samples, only 480 µL of the 500 µL were filtered to ensure the same filtered volume per well, avoiding volume variation problems after pipetting. Once the samples were pulled through the filter plate, each well was rinsed with 2 × 250 µL of complete assay buffer, filtered and dried before fluorescence measurements using a Victor X2 microplate fluorometer (PerkinElmer, Villebon-sur-Yvette, France). A 490/10 nm excitation filter and a 520/10 nm emission filter were used to obtain the relative fluorescence units (RFUs) for each well. The mean RFU value measured from the unused outer wells (*n* = 36) were subtracted from each well to account for any background fluorescence. The mean RFU value measured from the control wells (*n* = 8 in each experiment) was composed of Bodipy^®^-PbTx-2 + synaptosomes + assay buffer only and served as total binding reference (100%). Net fluorescence data were fitted to a sigmoidal dose-response curve (variable slope) based on the four-parameter logistic model (4PL) using GraphPad Prism v9.1.0 software (San Diego, CA, USA). From the sigmoidal curves, the effective concentrations inducing 80% and 50% of binding were determined as EC_80_ and EC_50_ for CTX3C, respectively, and expressed in pg mL^−1^. The EC_50_ of *Gambierdiscus* samples were expressed in cell mL^−1^.

#### 3.5.3. Solvent Effect

To evaluate if the presence of solvent may interfere in the response of the fRBA toward CTXs, eight-point serial 1:2 dilutions of EtOH, MeOH, and DMSO were tested. As described in Section 3.5.2, 20 µL of each concentration were tested in duplicate wells, with the first concentration composed of 20 µL of pure solvent, followed by seven concentrations diluted by a factor of 2 and incubated for 30 min at 37 °C.

#### 3.5.4. Incubation Parameters

In the initial protocols [116,136,143,149], the incubation step was set at 4°C for 90 min. Prior to the present study, temperature at 37 °C was also tested using six different incubation times: 10, 20, 30, 40, 50 and 60 min (data not shown). Thus, two incubation conditions were tested in two independent experiments on different days (inter-assay) comparing 4 °C for 90 min versus 37 °C for 30 min. For this experiment, an eight-point serial 1:2 dilution of *G. polynesiensis* TB92 strain was used with final concentrations ranging from 0.02 to 3 µg mL^−1^ of dry extracts corresponding to 16 to 2000 cell mL^−1^, with each concentration tested in duplicate wells for four independent experiments.

#### 3.5.5. Matrix Effects

The maximum concentration of *Gambierdiscus* dry extract (MCE) that does not induce unspecific binding was established using *G. pacificus* (TKR9) and *G. toxicus* (HPT3, RIK31, and TKU5) previously found negative by CBA-N2a (data not shown). These strains were extracted using the same chemical protocol [17]. Eight-point serial 1:2 dilutions were tested with eight concentrations ranging from 1.28 to 163, 1.8 to 230, 2.6 to 338 and 1.28 to 163 µg mL^−1^ for HPT3, RIK31, TKR9 and TKU5, respectively, corresponding to 500 to 64,000 cell mL^−1^ for HPT3, RIK31, and TKU5, and to 1000 to 128,000 cell mL^−1^ for TKR9, and incubated for 30 min at 37 °C.

The limit of detection (LOD) and the limit of quantification (LOQ) of fRBA applied to *Gambierdiscus* extracts were estimated using the following Equations (1) and (2):

LOD = (EC_80_ of CTX3C/MCE of *Gambierdiscus*) (1)

LOQ = (EC_50_ of CTX3C/MCE of *Gambierdiscus*) (2)

Both the LOD and LOQ were expressed in fg CTX3C eq cell^−1^.

The CTX content is the concentration of CTXs in each *Gambierdiscus* sample. This was estimated by comparing the EC_50_ value of CTX3C with the EC_50_ value of each tested *Gambierdiscus* sample and was further calculated using the following Equation (3):

CTX content = (EC_50_ of CTX3C/EC_50_ of *Gambierdiscus samples*) (3)

The CTX content was expressed in pg CTX3C equivalents (eq) cell^−1^.

#### 3.5.6. Repeatability of fRBA

To evaluate the repeatability of the fRBA, the DSF extracts of 30 *G. polynesiensis* samples were selected from the study of Longo, et al. [23] knowing their CTX concentration ranging from 0.47 to 4.23 pg CTX3C cell^−1^ as evaluated by LC-MS/MS from crude extracts (Table S1). To conduct the analyses using our fRBA protocol, 15 samples were tested at the same time in parallel with CTX3C, in three independent assays on different days and the same was performed for the other 15 remaining samples. An additional experiment was undertaken for some samples requiring range concentration adjustment. In the end, each sample was tested in *n* = 3 independent experiments and CTX3C in *n* = 2 × 3 + 1 = 7 independent experiments. The final concentrations of CTX3C ranged from 0.08 to 10 ng mL^−1^ and from 8 to 1000, 16 to 2000 or 32 to 4000 cell mL^−1^, according to the DSFs of *G. polynesiensis* samples, each concentration tested in triplicate wells, and incubated for 30 min at 37 °C.

### 3.6. Neuroblastoma Cell-Based Assay (CBA-N2a)

The CBA-N2a experiments were conducted following the procedure described previously [115]. The mouse neuroblastoma (N2a) cell line (CCL-131) was purchased at the American Type Culture Collection (ATCC, Manassas, VA, USA). In brief, a density of 50,000 ± 10,000 N2a cells/200 µL/well were seeded in 5% fetal bovine serum RPMI-1640 supplemented medium, in 96-well microtiter plates, and further kept at 37 °C in a humidified 5% CO_2_ atmosphere. For repeatability experiments, CTX3C and the 30 *G. polynesiensis* samples were tested in OV+ condition, only allowing for two samples to be analyzed per microplate. Thus after 24 h of growth (when the cell layer reached 100% confluence), the culture medium was replaced by 200 µL of 2% fetal bovine serum RPMI-1640 supplemented medium containing a non-destructive treatment of ouabain-veratridine (OV+) at a final concentration of 90/9 µM for all the internal wells. These treated N2a cells in OV+ conditions were exposed to a serial dilution 1:2 of eight concentrations of CTX3C or DSFs of *G. polynesiensis* samples. For the dry extracts of *Gambierdiscus* samples, the maximum concentration of dry extract (MCE) that does not induce unspecific mortalities in N2a cells was established at 9500 pg µL^−1^ corresponding to 1904 cell mL^−1^ [22]. Final concentrations of CTX3C ranged from 0.09 to 12.70 pg mL^−1^ and from 0.02 to 2.97 and from 0.18 to 23.81 cell mL^−1^, from the most toxic to the least potent sample, respectively. Each concentration was tested in triplicate wells per plate. Following an overnight incubation period, the viability of N2a cells was assessed using the MTT assay [115]. The absorbance was measured at 570 nm using a plate reader (iMark Microplate Absorbance Reader, BioRad, Marnes la Coquette, France). For all experiments, absorbance values of OV− and OV+ control wells were around 1.2, corresponding to 100% viability. Absorbance data were fitted to a sigmoidal dose–response curve (variable slope) based on the four-parameter logistic model (4PL) allowing for the calculation of EC_50_ values (i.e., the tested cell concentration inducing a loss of 50% of the higher absorbance value) in cell mL^−1^ using Prism v9.1.0 software (GraphPad, San Diego, CA, USA). The LOD and LOQ values of CBA-N2a and toxin content of *Gambierdiscus* samples were calculated using Equations (1)–(3). Fifteen samples were tested at the same time in parallel with CTX3C in three independent assays on different days, and the same was carried out for the 15 remaining samples. At the end, each sample was tested in *n* = 3 independent experiments and CTX3C in *n* = 2 × 3 = 6 independent experiments.

### 3.7. Liquid Chromatography Tandem Mass Spectrometry (LC-MS/MS)

In this study, one LC-MS/MS acquisition method was used to determine the toxin profile of the strains of *G. polynesiensis* for the P-CTX group based on the protocol method 2 by Sibat, et al. [21]. A brief description is given below.

Experiments were performed using a UHPLC system (UFLC Nexera, SHIMADZU, Kyoto, Japan) coupled to a hybrid triple quadrupole-linear ion-trap API4000 QTRAP mass spectrometer (SCIEX, Redwood city, CA, USA) equipped with a TurboV^®^ electrospray ionization source (ESI). The instrument control, data processing, and analysis were conducted using Analyst software 1.7.2 (Sciex, Framingham, MA, USA).

A linear gradient using water as eluent A and MeOH as eluent B, with both eluents containing 2 mM ammonium formate and 50 mM formic acid, was run on a Zorbax Eclipse Plus C18 column, 50 × 2.1 mm, 1.8 μm, 95 Å (Agilent Technologies, Santa Clara, CA, USA). The flow rate was 0.4 mL min^−1^, the injection volume was 5 μL, and the column temperature 40 °C. The elution gradient was as follows: 78% B to 88% B from 0 to 10 min, hold at 88% B for 4 min, decrease from 88% to 78% in 1 min and hold for 5 min at 78% B. The optimized ESI+ parameters were set as follows: curtain gas at 25 psi, ion spray at 5500 V, turbo gas temperature at 300 °C, gas 1 and 2 were set at 40 and 60 psi, respectively, and an entrance potential at 10 V.

Mass spectrometric detection was performed in positive ionization mode using the scheduled Multiple Reaction Monitoring (MRM) algorithm (Table S3). This algorithm optimizes dwell times and cycle time to provide better peak detection and improve reproducibility. A detection window of 90 s and a target scan time of 2 s were chosen for the MRM method. Calibration solution of CTX3C (ILM, Tahiti, French Polynesia) was prepared in MeOH, with concentration ranging from 10 to 1000 ng mL^−1^ in triplicates in three independent experiments (*n* = 3). In addition, a mix of P-CTX standards (ILM) were injected in each experiment to obtain a reference for the retention times. The MCE of *Gambierdiscus* matrix was established at 100,000 cell mL^−1^ and *G. polynesiensis* samples were tested at a concentration of 20,000 cell mL^−1^ in three independent experiments.

Quantitation of the different P-CTX analogs was performed using CTX3C (ILM) as a reference standard. Assuming an equal molar response and applying the same LOD and LOQ, the estimated concentrations of each CTX analog in *Gambierdiscus* sample were calculated against the CTX3C calibration curve and expressed in the equivalent CTX3C. The sum of the estimated concentrations of all CTX analogs allowed for an estimation of the total CTX content (expressed in pg CTX3C eq cell^−1^). All 30 *Gambierdiscus* samples were analyzed in three independent experiments on different days (*n* = 3).

### 3.8. Statistical Analyses

To compare fRBA incubation conditions, EC_50_ values generated at different temperatures and incubation times were analyzed using a paired *t*-test (GraphPad Prism v.7.05, GraphPad Software, San Diego, CA, USA). To compare strain differences using individual assays, one-way ANOVAs were conducted with Tukey’s multiple comparisons posthoc analysis (GraphPad Prism v.7.05).

Descriptive statistics were performed to calculate the mean ± standard deviation (SD) of EC_50_ values and CTX contents obtained from three independent experiments on different days of each sample tested by each assay.

The coefficient of variation (CV) is defined as the ratio of the standard deviation to the mean and calculated according to the following Equation (4):

CV = (SD/mean) × 100(4)

The CV (also known as the relative standard deviation, RSD) is expressed in percentages (%) and is a statistical measure of the dispersion of data points around the mean, used to express the precision and repeatability of an assay.

The distribution of CTX content for each assay was graphed using the histogram analysis in GraphPad Prism v.7.05. To determine the degree to which the different assays were correlated with each other, Pearson’s correlation analyses were performed to assess the correlation coefficient (*r*^2^) (GraphPad Prism v.7.05). To assess if the assays yielded significantly different estimates of toxicity, repeated measures ANOVAs were performed on individual sample with Tukey’s multiple comparisons posthoc analysis (GraphPad Prism v.7.05). Comparisons were considered significant if a *p*-value of less than 0.05 was obtained.

## 4. Conclusions

In this study, the fRBA was applied for the first time to *Gambierdiscus* matrix, showing its good potential for the detection of algal CTXs. Our intra-laboratory validation study highlights the great performance of fRBA, CBA-N2a, and LC-MS/MS to detect and quantify CTXs in *Gambierdiscus* matrix with regards to the sensitivity, specificity, and repeatability of these methods. Additionally, significant correlations were observed between the three methods, especially between fRBA and LC-MS/MS. Of the three assays, CBA-N2a was the most sensitive. It is also noteworthy that, despite using the same CTX3C standard and chemical extraction method, differences were observed in CTX quantification between the three methods used in some samples. The CTX quantification obtained by these three methods also confirmed the existence of an intraspecific variability in CTX production in *G. polynesiensis* under different culturing conditions.

Currently, based on the European Union legal regulations, CTXs are still regarded as a poorly regulated group of marine toxins due to (i) the lack of a reference detection method duly validated at an international level, (ii) the lack of or poor availability of commercial or certified CTX standards, as well as biological reference materials (positive and negative samples), and (iii) the absence of clearly defined regulatory thresholds [150,151]. At present, among the three methods evaluated in this study, only CBA-N2a has been the subject of a collaborative study between five laboratories for the detection of three families of lipophilic toxins, i.e., azaspiracids, pectenotoxins, and okadaic acid [152,153], while a radio-labelled version of the receptor-binding assay and ultra-high-performance hydrophilic interaction liquid chromatography with tandem mass spectrometry have also undergone inter-laboratory validation for saxitoxins and/or tetrodotoxins [131,154]. Based on the findings of the present study, an inter-laboratory follow-up study would be a logical step forward since the standardization of extraction protocols and detection methods for CTXs has yet to be undertaken. Indeed, the harmonization and validation of reference methods for CTXs will facilitate a more rigorous comparison of ciguatoxicity data of CTX-producing species between ciguatera-endemic regions, and hence allow for a more reliable assessment of ciguatera risk worldwide.

## Figures and Tables

**Table 1:** Comparison of the performances of the fluorescent receptor binding assay (fRBA) with published studies for fRBA and radioactive receptor binding assay (rRBA) for the detection of ciguatoxins in *Gambierdiscus* matrix.

Toxin and Matrix	Parameters ^1^	fRBA		rRBA		
References		This Study	[136]	[87,88,126]	[91]	[84]
CTX3C	LOD = EC_80_ (ng mL^−1^)	1.10 ± 0.08	ND ^2^	0.90 ± 0.19 ^3^	ND	ND
	LOQ = EC_50_ (ng mL^−1^)	2.10 ± 0.16	0.66 ± 0.16	1.92 ± 0.38 ^3^	0.62 ± 0.13	0.62 ± 0.16
	EC_20_ (ng mL^−1^)	4.06 ± 0.59	ND	4.17 ± 1.12 ^3^	ND	ND
*Gambierdiscus*	MCE (cell mL^−1^)	16,000	ND	7500	ND	ND
	LOD (fg CTX3C eq cell^−1^)	68.53 ± 5.25	ND	250	ND	15.5
	LOQ (fg CTX3C eq cell^−1^)	131.32 ± 9.94	ND	ND	ND	<310–330

^1^ LOD = limit of detection, LOQ = limit of quantification, MCE = maximum concentration of matrix that does not induce unspecific effects with all values presented as the mean ± standard deviation (SD). ^2^ ND: no data available. ^3^ Data of CTX3C (Wako origin) were converted in ng mL^−1^ originally expressed in nanomolar (nM) in Díaz-Asencio, et al. [126].

**Table 2:** Composite binding affinity and CTX contents in 30 *Gambierdiscus polynesiensis* samples as estimated by the fluorescent receptor binding assay (fRBA).

Strain Name ^1^	ID#	EC_50_^2^ (cell mL^−1^)	Coefficient of Variation (CV %) ^3^	CTX Content ^2^ (pg CTX3C eq cell^−1^)	Coefficient of Variation (CV %) ^3^
RG92	RG92-a	1188 ± 563	51	2.04 ± 1.40	69
	RG92-b	1926 ± 237	12	1.10 ± 0.15	14
	RG92-c	1314 ± 101	8	1.64 ± 0.17	10
RIK7	RIK7-a	482 ± 41	9	4.36 ± 0.14	3
	RIK7-b	467 ± 64	14	4.54 ± 0.48	11
	RIK7-c	747 ± 36	5	2.88 ± 0.23	8
	RIK7-d	1006 ± 57	6	2.14 ± 0.13	6
	RIK7-e	448 ± 26	6	4.70 ± 0.18	4
	RIK7-f	1143 ± 89	8	1.89 ± 0.31	16
	RIK7-g	387 ± 14	4	5.56 ± 0.64	11
	RIK7-h	405 ± 20	5	5.19 ± 0.21	4
	RIK7-i	794 ± 85	11	2.74 ± 0.50	18
	RIK7-j	368 ± 24	6	5.87 ± 0.76	13
	RIK7-k	561 ± 47	8	3.76 ± 0.53	14
	RIK7-l	415 ± 51	12	5.07 ± 0.49	10
RAI1	RAI1-a	439 ± 43	10	4.90 ± 0.10	2
	RAI1-b	439 ± 33	8	4.92 ± 0.66	13
	RAI1-c	377 ± 28	7	5.73 ± 0.73	13
	RAI1-d	442 ± 30	7	4.88 ± 0.50	10
	RAI1-e	376 ± 30	8	5.60 ± 0.35	6
	RAI1-f	466 ± 230	49	5.53 ± 2.12	38
	RAI1-g	579 ± 33	6	3.63 ± 0.45	12
	RAI1-h	360 ± 14	4	5.87 ± 0.73	13
NHA4	NHA4-a	267 ± 12	5	8.05 ± 0.40	5
	NHA4-b	255 ± 16	6	8.27 ± 0.68	8
	NHA4-c	270 ± 15	6	7.97 ± 0.76	10
	NHA4-d	272 ± 19	7	7.72 ± 0.17	2
	NHA4-e	279 ± 15	5	7.53 ± 0.35	5
	NHA4-f	279 ± 8	3	7.70 ± 0.46	6
	NHA4-g	263 ± 11	4	8.01 ± 0.65	8

^1^ All strains were cultured under urea as nitrogen source, different N/P ratio and pH values for 21 or 30 days from the study of Longo, et al. [23]. ^2^ The presented values corresponded to the mean ± standard deviation (SD) from three independent experiments (*n* = 3). ^3^ The coefficients of variation (CV) were calculated according to Equation (4) (see Section 3.8).

**Table 3:** Comparison of the performances of the neuroblastoma cell-based assay (CBA-N2a) with published studies for CBA-N2a for the detection of ciguatoxins in *Gambierdiscus* matrix.

OV Treatment	CTX3C ^1^		*Gambierdiscus* Matrix ^1^			
O/V (µM)	LOD = EC_80_ (pg mL^−1^)	LOQ = EC_50_ (pg mL^−1^)	MCE (cell mL^−1^)	LOD (fg CTX3C eq cell^−1^)	LOQ (fg CTX3C eq cell^−1^)	References
76.2/7.62	ND ^2^	ND	ND	0.17	0.34	[22]
80/8	ND	3.10 ± 0.76	ND	0.17	ND	[19,140]
(80/8–90/9)	ND	1.91 ± 0.22	ND	ND	ND	[89]
90/9	0.63 ± 0.05	1.50 ± 0.23	1904	0.33 ± 0.03	0.79 ± 0.12	This study
100/10	ND	ND	ND	ND	8.7 × 10^−4^–4.7 × 10^−2^	[93] ^3^
	ND	ND	ND	ND	6.74–7.27 × 10^−7^	[92] ^3^
	0.2	ND	ND	ND	ND	[94]
	ND	1.44 ± 0.70	ND	0.17	ND	[96]
	ND	1.73 ± 0.41	ND	ND	ND	[115]
	ND	1.84 ± 0.31	ND	ND	N−D	[109]
	ND	3.52 ± 0.27	ND	ND	ND	[139]
250/25	ND	1.66 ± 0.16	ND	ND	ND	[136]
	ND	1.66 ± 0.16	ND	ND	ND	[95]
500/50	ND	0.91 ± 0.13	ND	ND	ND	[138]
	ND	1.30 ± 0.06	ND	ND	ND	[91]
	ND	0.57 ± 0.11	ND	ND	ND	[137]

^1^ LOD = limit of detection, LOQ = limit of quantification, MCE = maximum concentration of matrix that does not induce unspecific effects, with all values presented as the mean ± standard deviation (SD). ^2^ ND: no data available. ^3^ LOQ values was expressed in pg CTX1 eq cell^−1^ and could be divided by 0.2 for CTX3C correspondence, as recommended by the authors [92].

**Table 4:** Composite cytotoxicity and CTX contents in 30 *Gambierdiscus polynesiensis* samples as estimated by the neuroblastoma cell-based assay (CBA-N2a).

Strain Name ^1^	ID#	EC_50_^2^ (cell mL^−1^)	Coefficient of Variation (CV %) ^3^	CTX Content ^2^ (pg CTX3C eq cell^−1^)	Coefficient of Variation (CV %) ^3^
RG92	RG92-a	2.28 ± 0.54	24%	0.68 ± 0.09	14
	RG92-b	2.08 ± 0.46	23%	0.68 ± 0.03	4
	RG92-c	2.15 ± 0.50	22%	0.75 ± 0.10	14
RIK7	RIK7-a	0.73 ± 0.18	25%	2.03 ± 0.12	6
	RIK7-b	0.67 ± 0.10	15%	2.19 ± 0.30	14
	RIK7-c	0.76 ± 0.09	12%	2.03 ± 0.35	17
	RIK7-d	1.45 ± 0.39	27%	1.10 ± 0.35	22
	RIK7-e	0.59 ± 0.13	23%	2.53 ± 0.54	21
	RIK7-f	0.72 ± 0.09	12%	2.14 ± 0.17	8
	RIK7-g	0.66 ± 0.13	20%	2.34 ± 0.32	14
	RIK7-h	0.45 ± 0.14	30%	3.39 ± 0.91	27
	RIK7-i	1.96 ± 0.31	16%	0.79 ± 0.08	10
	RIK7-j	0.46 ± 0.08	17%	3.33 ± 0.43	13
	RIK7-k	0.64 ± 0.16	25%	2.32 ± 0.36	16
	RIK7-l	0.45 ± 0.13	29%	3.31 ± 0.33	10
RAI1	RAI1-a	0.65 ± 0.17	26%	2.44 ± 0.48	20
	RAI1-b	0.60 ± 0.11	18%	2.57 ± 0.31	12
	RAI1-c	0.65 ± 0.10	15%	2.38 ± 0.41	22
	RAI1-d	0.43 ± 0.07	16%	3.55 ± 0.16	5
	RAI1-e	0.50 ± 0.14	28%	3.00 ± 0.24	8
	RAI1-f	0.55 ± 0.09	17%	2.70 ± 0.35	13
	RAI1-g	0.64 ± 0.13	20%	2.31 ± 0.16	7
	RAI1-h	0.31 ± 0.04	14%	4.72 ± 0.31	7
NHA4	NHA4-a	0.31 ± 0.06	19%	5.04 ± 0.63	13
	NHA4-b	0.21 ± 0.06	27%	6.93 ± 0.66	10
	NHA4-c	0.28 ± 0.06	20%	5.75 ± 0.92	16
	NHA4-d	0.36 ± 0.14	38%	4.21 ± 0.73	17
	NHA4-e	0.33 ± 0.05	16%	4.45 ± 0.86	19
	NHA4-f	0.40 ± 0.06	16%	3.90 ± 0.49	13
	NHA4-g	0.15 ± 0.04	29%	9.72 ± 1.64	17

^1^ All strains were cultured under urea as a nitrogen source, different N/P ratio and pH values for 21 or 30 days from the study of Longo, et al. [23]. ^2^ The indicated values correspond to the mean ± standard deviation (SD) from three independent experiments (*n* = 3). ^3^ The coefficients of variation (CV) were calculated according to Equation (4) (see Section 3.8).

**Table 5:** Comparison of the performances of the liquid chromatography tandem mass spectrometry (LC-MS/MS) with published studies for LC-MS/MS for the detection of ciguatoxins in *Gambierdiscus* matrix.

CTX3C ^1^		*Gambierdiscus* ^1^			References
LOD (ng mL^−1^)	LOQ (ng mL^−1^)	MCE (cell mL^−1^)	LOD (fg CTX3C eq cell^−1^)	LOQ (fg CTX3C eq cell^−1^)	
2	6	100,000	20	60	This study
5	10	ND ^2^	ND	ND	[23]
2	6	ND	ND	ND	[109]
ND	40	ND	ND	[20–80]	[22]
1	ND	ND	ND	ND	[8,106]
60	ND	ND	ND	ND	[19]
1–2	ND	ND	ND	ND	[104]
ND	ND	ND	0.005	ND	[76]

^1^ LOD = limit of detection, LOQ = limit of quantification, MCE = maximum concentration of matrix that does not induce unspecific effects. ^2^ ND: no data available.

**Table 6:** Mean CTX contents in 30 *Gambierdiscus polynesiensis* samples as estimated by liquid chromatography tandem mass spectrometry (LC-MS/MS).

Strain Name ^1^	ID#	CTX3B ^2^	CTX3C	CTX4A	CTX4B	M-Seco-CTX3C	2OH-CTX3C	3OH-CTX3C	CTX3C Isomers ^4^					Total CTX Content ^2^ (pg CTX3C eq cell^−1^)	Coefficient of Variation (CV %) ^5^
									1 (6.90)	2 (7.25)	3 (7.70)	4 (9.80)	5 (10.10)		
RG92	RG92-a	0.35 ± 0.01	0.23 ± 0.02	0.07 ± 0.02	0.09 ± 0.02	<LOD ^3^	0.09 ± 0.00	<LOD	0.06 ± 0.03	0.05 ± 0.03	0.06 ± 0.00	0.06 ± 0.04	0.07 ± 0.01	1.03 ± 0.10	10%
	RG92-b	0.41 ± 0.04	0.42 ± 0.11	0.05 ± 0.01	0.07 ± 0.01	<LOD	<LOD	<LOD	<LOQ ^3^	<LOQ	0.05 ± 0.03	<LOD	0.06 ± 0.04	1.01 ± 0.14	14%
	RG92-c	0.43 ± 0.01	0.47 ± 0.02	0.04 ± 0.04	0.10 ± 0.02	<LOD	<LOD	<LOD	0.05 ± 0.03	0.05 ± 0.03	0.06 ± 0.00	<LOD	0.07 ± 0.01	1.24 ± 0.06	5%
RIK7	RIK7-a	1.33 ± 0.17	0.96 ± 0.13	0.05 ± 0.01	0.07 ± 0.01	<LOD	0.11 ± 0.01	<LOD	0.05 ± 0.03	0.06 ± 0.03	0.07 ± 0.04	0.07 ± 0.04	0.09 ± 0.00	2.76 ± 0.31	11%
	RIK7-b	1.51 ± 0.02	1.12 ± 0.11	0.06 ± 0.01	0.09 ± 0.03	<LOD	0.11 ± 0.01	<LOD	0.06 ± 0.03	0.07 ± 0.05	0.07 ± 0.02	0.08 ± 0.00	0.08 ± 0.01	3.18 ± 0.18	6%
	RIK7-c	0.98 ± 0.07	0.80 ± 0.04	0.10 ± 0.09	0.24 ± 0.07	<LOD	<LOD	<LOD	0.05 ± 0.03	0.06 ± 0.03	0.06 ± 0.00	<LOD	0.06 ± 0.04	2.27 ± 0.20	9%
	RIK7-d	0.98 ± 0.06	0.56 ± 0.01	0.05 ± 0.04	0.09 ± 0.02	<LOD	<LOD	<LOD	<LOQ	0.06 ± 0.00	0.05 ± 0.03	<LOD	0.07 ± 0.04	1.81 ± 0.16	9%
	RIK7-e	2.81 ± 0.21	1.42 ± 0.22	0.15 ± 0.03	0.23 ± 0.07	<LOD	<LOD	0.05 ± 0.03	0.06 ± 0.03	0.08 ± 0.01	0.07 ± 0.01	0.07 ± 0.04	0.07 ± 0.04	4.90 ± 0.48	10%
	RIK7-f	1.37 ± 0.06	0.98 ± 0.06	0.15 ± 0.08	0.26 ± 0.07	<LOD	0.05 ± 0.00	<LOD	0.06 ± 0.03	0.07 ± 0.01	0.08 ± 0.01	0.06 ± 0.04	0.08 ± 0.05	3.08 ± 0.07	2%
	RIK7-g	1.75 ± 0.03	1.31 ± 0.08	0.20 ± 0.12	0.43 ± 0.15	<LOD	0.06 ± 0.00	<LOD	0.06 ± 0.03	0.09 ± 0.01	0.10 ± 0.01	0.06 ± 0.01	0.07 ± 0.01	4.11 ± 0.16	4%
	RIK7-h	3.16 ± 0.25	1.58 ± 0.17	0.13 ± 0.03	0.18 ± 0.06	<LOD	<LOD	<LOD	<LOQ	0.07 ± 0.02	0.07 ± 0.02	0.06 ± 0.03	0.07 ± 0.04	5.25 ± 0.49	9%
	RIK7-i	0.92 ± 0.03	0.57 ± 0.03	0.06 ± 0.06	0.15 ± 0.04	<LOD	<LOD	<LOD	0.05 ± 0.03	0.05 ± 0.03	0.06 ± 0.01	<LOD	0.06 ± 0.04	1.87 ± 0.10	6%
	RIK7-j	3.07 ± 0.08	1.71 ± 0.21	0.10 ± 0.04	0.17 ± 0.04	<LOD	<LOD	0.06 ± 0.04	0.06 ± 0.03	0.11 ± 0.00	0.13 ± 0.01	0.07 ± 0.04	0.07 ± 0.04	5.41 ± 0.24	4%
	RIK7-k	1.96 ± 0.08	1.23 ± 0.17	0.19 ± 0.04	0.30 ± 0.09	<LOD	0.06 ± 0.03	<LOD	<LOQ	0.05 ± 0.03	0.07 ± 0.04	0.06 ± 0.03	0.06 ± 0.04	3.82 ± 0.35	9%
	RIK7-l	3.26 ± 0.10	1.65 ± 0.09	0.14 ± 0.03	0.22 ± 0.06	0.05 ± 0.03	<LOD	<LOD	0.06 ± 0.03	0.08 ± 0.01	0.10 ± 0.01	0.05 ± 0.03	0.06 ± 0.04	5.56 ± 0.12	2%
RAI1	RAI1-a	1.81 ± 0.02	1.29 ± 0.04	0.09 ± 0.03	0.17 ± 0.05	<LOD	0.06 ± 0.00	<LOD	0.06 ± 0.01	0.10 ± 0.02	0.10 ± 0.02	0.07 ± 0.01	0.08 ± 0.01	3.84 ± 0.01	0%
	RAI1-b	1.43 ± 0.04	1.10 ± 0.07	0.12 ± 0.05	0.19 ± 0.05	<LOD	0.10 ± 0.00	<LOD	0.05 ± 0.03	0.08 ± 0.01	0.13 ± 0.01	0.07 ± 0.01	0.07 ± 0.01	3.33 ± 0.05	2%
	RAI1-c	1.46 ± 0.08	1.32 ± 0.01	0.20 ± 0.14	0.42 ± 0.13	<LOD	0.09 ± 0.00	<LOD	0.06 ± 0.03	0.10 ± 0.01	0.11 ± 0.01	0.07 ± 0.01	0.07 ± 0.00	3.87 ± 0.28	7%
	RAI1-d	2.73 ± 0.03	1.75 ± 0.17	0.12 ± 0.05	0.27 ± 0.08	<LOD	<LOD	<LOD	0.05 ± 0.03	0.07 ± 0.01	0.08 ± 0.01	0.06 ± 0.03	0.06 ± 0.04	5.12 ± 0.16	3%
	RAI1-e	2.91 ± 0.18	2.12 ± 0.35	0.10 ± 0.02	0.17 ± 0.05	<LOD	<LOD	<LOD	0.05 ± 0.03	0.10 ± 0.02	0.12 ± 0.01	<LOQ	0.08 ± 0.05	5.60 ± 0.52	9%
	RAI1-f	1.40 ± 0.02	0.92 ± 0.06	0.08 ± 0.02	0.09 ± 0.03	<LOD	<LOD	<LOD	<LOQ	0.06 ± 0.03	0.05 ± 0.03	0.05 ± 0.03	0.07 ± 0.04	2.60 ± 0.14	6%
	RAI1-g	1.76 ± 0.08	1.22 ± 0.11	0.15 ± 0.04	0.23 ± 0.07	<LOD	0.05 ± 0.03	<LOQ	0.06 ± 0.03	0.06 ± 0.01	0.09 ± 0.01	0.05 ± 0.03	0.06 ± 0.04	3.61 ± 0.27	7%
	RAI1-h	3.28 ± 0.15	1.91 ± 0.15	0.15 ± 0.03	0.22 ± 0.06	<LOD	<LOD	<LOD	0.06 ± 0.03	0.10 ± 0.02	0.11 ± 0.00	0.06 ± 0.01	0.08 ± 0.05	5.91 ± 0.32	5%
NHA4	NHA4-a	3.74 ± 0.14	2.37 ± 0.10	0.07 ± 0.01	0.11 ± 0.02	0.10 ± 0.01	<LOD	<LOD	0.06 ± 0.03	0.14 ± 0.01	0.16 ± 0.01	0.07 ± 0.01	0.07 ± 0.01	6.84 ± 0.21	3%
	NHA4-b	4.12 ± 0.19	2.44 ± 0.33	0.08 ± 0.01	0.12 ± 0.04	0.07 ± 0.01	<LOQ	0.07 ± 0.00	<LOQ	0.08 ± 0.03	0.10 ± 0.01	0.05 ± 0.03	0.07 ± 0.04	7.14 ± 0.51	7%
	NHA4-c	4.31 ± 0.14	2.14 ± 0.16	0.08 ± 0.01	0.10 ± 0.02	0.09 ± 0.01	<LOD	0.06 ± 0.00	0.06 ± 0.01	0.14 ± 0.02	0.14 ± 0.01	0.06 ± 0.01	0.07 ± 0.01	7.25 ± 0.10	1%
	NHA4-d	3.27 ± 0.10	2.53 ± 0.17	0.10 ± 0.02	0.18 ± 0.05	0.11 ± 0.01	0.11 ± 0.01	<LOD	<LOQ	0.13 ± 0.07	0.17 ± 0.02	0.07 ± 0.02	0.08 ± 0.05	6.68 ± 0.19	3%
	NHA4-e	4.32 ± 0.19	2.72 ± 0.50	0.08 ± 0.01	0.11 ± 0.03	0.11 ± 0.00	0.05 ± 0.03	<LOD	<LOQ	0.12 ± 0.01	0.17 ± 0.03	0.06 ± 0.00	0.07 ± 0.01	7.76 ± 0.70	9%
	NHA4-f	3.66 ± 0.11	2.17 ± 0.07	0.09 ± 0.02	0.12 ± 0.03	0.09 ± 0.01	0.05 ± 0.00	0.06 ± 0.03	0.06 ± 0.04	0.16 ± 0.02	0.21 ± 0.01	0.06 ± 0.01	0.07 ± 0.01	6.74 ± 0.10	1%
	NHA4-g	4.87 ± 0.18	2.67 ± 0.25	0.09 ± 0.03	0.12 ± 0.04	0.09 ± 0.01	<LOD	0.05 ± 0.03	<LOQ	0.14 ± 0.02	0.18 ± 0.02	0.06 ± 0.03	0.07 ± 0.04	8.25 ± 0.45	6%

^1^ All strains were cultured under urea as nitrogen source, different N/P ratio and pH values for 21 or 30 days (Table S2) as described in the study of Longo, et al. [23]. ^2^ The values correspond to the mean ± standard deviation (SD) from three independent experiments (*n* = 3). ^3^ LOD= Limit of detection and LOQ = Limit of quantification. ^4^ Retention time (min) of each isomers are shown in brackets. ^5^ The coefficients of variation (CV) of total CTX contents were calculated according to Equation (4) (see Section 3.8).

**Table 7:** Summary of statistics (minimum, maximum, mean, median and standard deviation of mean) obtained from CTX quantification of *Gambierdiscus* matrix using the fluorescent binding assay (fRBA), the neuroblastoma cell-based assay (CBA-N2a), and the liquid chromatography tandem mass spectrometry (LC-MS/MS).

Method	Min	Max	Median	Mean	Standard Deviation of Mean
fRBA	1.10	8.27	5.002	4.996	2.11
CBA-N2a	0.68	9.72	2.553	3.103	1.94
LC-MS/MS	1.01	8.25	3.990	4.395	2.10

## Data Availability

Not applicable.

## Supplementary Materials

The following supporting information can be downloaded at: https://www.mdpi.com/article/10.3390/md20060348/s1. Table S1: Geographic origin and year of isolation of *Gambierdiscus strains* selected for this study; Table S2: Culture conditions and CTX contents estimated on crude extracts using the liquid chromatography tandem mass spectrometry (LS-MS/MS) of the 30 *Gambierdiscus polynesiensis* samples [23]; Table S3: Selected *m*/*z* transitions and liquid chromatography tandem mass spectrometry (LC-MS/MS) instrument parameters used for the scheduled MRM method.; Figure S1: Binding dose–response curves of ethanol (●), methanol (■) and DMSO (▲) using the fluorescent receptor binding assay (fRBA) incubated at 37 °C for 30 min. Data represent the mean ± standard deviation (SD) of one experiment, each concentration run in duplicate wells; Figure S2: Binding dose–response curves of *Gambierdiscus pacificus* (TKR9 ■) and *G. toxicus* (RIK31●, HPT3 ◊ and TKU5 ▲) samples using the fluorescent receptor binding assay (fRBA) incubated at 37 °C for 30 min. Matrix effects of four *Gambierdiscus* strains with their concentrations expressed (a) in µg of dry extract mL^−1^ and (b) in cell mL^−1^. Data represent the mean ± standard deviation (SD) of one experiment, each concentration run in duplicate wells. The dotted vertical lines correspond to the maximum concentration of *Gambierdiscus* extract equivalent to MCE = 55 ± 26 µg of dry extract mL^−1^ corresponding to 16,000 cell mL^−1^ for matrix interference.
