# Supplementary material for: Comparative Study on the Performance of Three Detection Methods for the Quantification of Pacific Ciguatoxins in French Polynesian Strains of Gambierdiscus polynesiensis
Source: Mar Drugs. 2022 May 25;20(6):348. doi: 10.3390/md20060348 (PMC9229625; doi:10.3390/md20060348)
Supplement: Supplementary file 1 [file marinedrugs-20-00348-s001.zip › marinedrugs-1728436-supplementary.pdf]

marine drugs

Supplementary Materials :
Comparative Study on the Performance of Three Detection Methods for the Quantification of Pacific Ciguatoxins in French Polynesian Strains of *Gambierdiscus polynesiensis*

Hélène Taiana Darius[1]*, Taina Revel[1], Jérôme Viallon[1], Manoëlla Sibat[2], Philippe Cruchet[1], Sébastien Longo[1], D. Ransom Hardison[3], William C. Holland[3], Patricia A. Tester[4], R. Wayne Litaker[5], Jennifer R. McCall[6], Philipp Hess[2], Mireille Chinain[1].

[1] Institut Louis Malardé (ILM), Laboratory of Marine Biotoxins, UMR 241-EIO (IFREMER, ILM, IRD, Université de Polynésie Française), P.O. Box 30, Papeete 98713, French Polynesia; trevel@ilm.pf (T.R.); jviallon@ilm.pf (J.V.); pcruchet@ilm.pf (P.C.); sebastien.longo81@gmail.com (SL); mchinain@ilm.pf (M.C.)
[2] IFREMER, PHYTOX, Laboratoire METALG, F-44000 Nantes, France ; manoella.sibat@ifremer.fr (M.S.); philipp.hess@ifremer.fr (P.H.)
[3] National Oceanic and Atmospheric Administration, Center for Coastal Fisheries and Habitat Research, Beaufort, North Carolina, United States of America; rance.hardison@noaa.gov (D.R.H.); chris.holland@noaa.gov (W.C.H.)
[4] Ocean Tester, LLC, Beaufort, North Carolina, United States of America; ocean.tester@gmail.com (P.A.T.)
[5] CSS, Inc. under contract to National Oceanic and Atmospheric Administration, National Centers for Coastal Ocean Science, National Ocean Service, Beaufort, NC 28516, USA; wayne.r.litaker@noaa.gov (R.W.L.)
[6] Center for Marine Science, University of North Carolina Wilmington, 601 South College Road, Wilmington, NC 28403-5915; mccalljr@uncw.edu (J.R.M.)
* Correspondence: tdarius@ilm.pf; Tel.: +689-40-416-484

Table S1. Geographic origin and year of isolation of *Gambierdiscus strains* selected for this study.

Taxonomic identity	Strain ID	Year of isolation	Archipelago	Island
Gambierdiscus pacificus	TKR9	2016	Society	Tahiti
Gambierdiscus toxicus	HPT3	2016	Society	Moorea
	RIK31	2017	Gambier	Mangareva
	TKU5	2017	Gambier	Mangareva
Gambierdiscus polynesiensis	TB92	1992	Australes	Tubuai
	RG92	1992	Tuamotu	Rangiroa
	RAI1	2008	Australes	Raivavae
	RIK7	2013	Gambier	Mangareva
	NHA4	2015	Marquesas	Nuku Hiva

Table S2. Culture conditions and CTX contents estimated on crude extracts using the liquid chromatography tandem mass spectrometry (LS-MS/MS) in the 30 *Gambierdiscus polynesiensis* samples.

Strain name[1]	Sample ID#	N/P ratio	pH	Day of culture*	Biomass (cells)	CTX content (pg CTX3C eq. cell^{-1})
RG92	RG92-a	48	8.2	21	79,772	0.47
	RG92-b	48	8.2	30	119,500	0.61
	RG92-c	48	8.2	30	128,825	0.67
RIK7	RIK7-a	48	7.9	30	84,730	1.00
	RIK7-b	48	7.9	30	80,216	1.20
	RIK7-c	48	8.4	21	112,161	1.22
	RIK7-d	48	8.4	21	106,722	1.35
	RIK7-e	24	8.4	30	84,075	1.67
	RIK7-f	48	8.2	21	84,060	1.73
	RIK7-g	48	8.2	21	92,316	1.94
	RIK7-h	24	8.4	30	118,625	1.96
	RIK7-i	48	8.2	30	114,000	2.03
	RIK7-j	48	8.4	30	133,650	2.10
	RIK7-k	48	8.2	30	140,675	2.11
	RIK7-l	48	8.4	30	122,775	2.33
RAI1	RAI1-a	48	7.9	30	94,967	1.76
	RAI1-b	48	8.2	30	98,725	1.76
	RAI1-c	48	7.9	30	81,597	1.94
	RAI1-d	48	8.4	21	98,123	1.96
	RAI1-e	48	8.4	30	137,700	2.02
	RAI1-f	48	8.4	21	84,305	2.03
	RAI1-g	48	8.2	30	117,825	2.23
	RAI1-h	48	8.4	30	127,400	2.53
NHA4	NHA4-a	48	8.4	30	173,150	2.52
	NHA4-b	48	8.4	21	79,282	2.96
	NHA4-c	48	8.4	30	115,825	3.20
	NHA4-d	48	8.2	30	117,275	3.55
	NHA4-e	48	8.2	30	133,625	3.55
	NHA4-f	48	8.2	21	85,946	4.23
	NHA4-g	48	8.2	21	94,644	4.23

[1]All strains were cultured under urea as nitrogen source, different N/P ratio and pH values during 21 or 30 days during the study of Longo, et al. [23].

Table S3. Selected m/z transitions and liquid chromatography tandem mass spectrometry (LC-MS/MS) instrument parameters used for the scheduled MRM method.

**Compound**	**Detection window** (min)	**Precursor ion** (Q1) *m/z*	**Product ion** (Q3) *m/z*	**DP** (eV)	**CE** (eV)	**CXP** (eV)
CTX1B, CTX1A	3.1 ± 1.5	1128.6 $[M+NH_4]^+$	1093.6	105	20	12
			1075.6	105	30	12
			95.1	105	90	20
M-*seco*-CTX3C	4.7 ± 1.5	1041.6 $[M+H]^+$	1023.6	105	30	12
			1005.6	105	20	12
			125.1	105	50	18
2,3-dihydro-2-hydroxyCTX3C and 2,3-dihydro-3-hydroxyCTX3C	5.4 ± 1.5	1058.6 $[M+NH_4]^+$	1023.6	105	30	12
			1005.6	105	20	12
			125.1	105	50	18
2,3-dihydro-2,3-dihydroxyCTX3C	6.0 ± 1.5	1074.6 $[M+NH_4]^+$	1039.6	105	30	12
		1057.6 $[M+H]^+$	1039.6	105	20	12
			125.1	105	50	18
51-hydroxyCTX3C	6.3 ± 1.5	1056.6 $[M+NH_4]^+$	1021.6	105	30	12
		1039.6 $[M+H]^+$	1021.6	105	20	12
			1003.6	105	20	12
M-*seco*-CTX4A/4B	6.5 ± 1.5	1096.6 $[M+NH_4]^+$	1043.7	105	30	12
		1079.6 $[M+H]^+$	1043.7	105	20	12
			125.1	105	50	18
52-*epi*-54-deoxyCTX1B and 54-deoxyCTX1B	6.8 ± 1.5	1112.6 $[M+NH_4]^+$	1077.6	105	20	12
			1059.6	105	30	12
			95.1	105	90	20
CTX3C isomers (1), (2) and (3)	7.6 ± 1.5	1040.6 $[M+NH_4]^+$	1005.6	105	30	12
		1023.6 $[M+H]^+$	1005.6	105	20	12
			125.1	105	20	12
CTX3C, CTX3B and isomers (4) and (5)	10.5 ± 1.5	1040.6 $[M+NH_4]^+$	1005.6	105	30	12
		1023.6 $[M+H]^+$	1005.6	105	20	12
			125.1	105	50	18
CTX4A and CTX4B	12.2 ± 1.5	1078.6 $[M+NH_4]^+$	1043.6	105	30	12
		1061.6 $[M+H]^+$	1043.6	105	20	12
			125.1	105	50	18

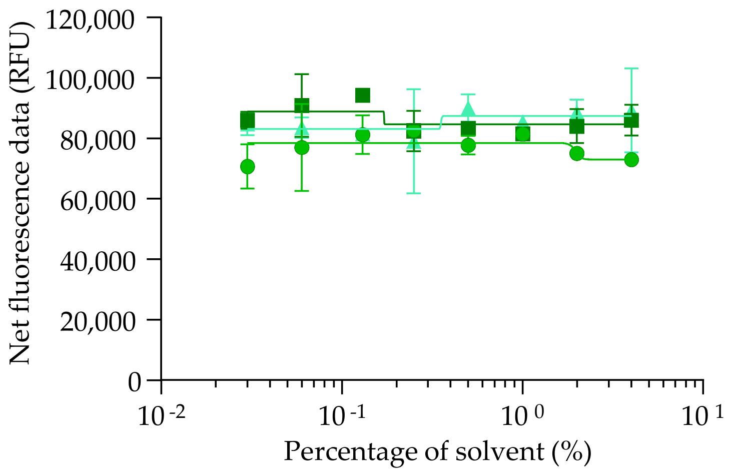

Figure S1. Binding dose-response curves of ethanol (●), methanol (■) and DMSO (▲) using the fluorescent receptor binding assay (fRBA) incubated at 37°C during 30 min. Data represent the mean ± standard deviation (SD) of one experiment, each concentration run in duplicate wells.

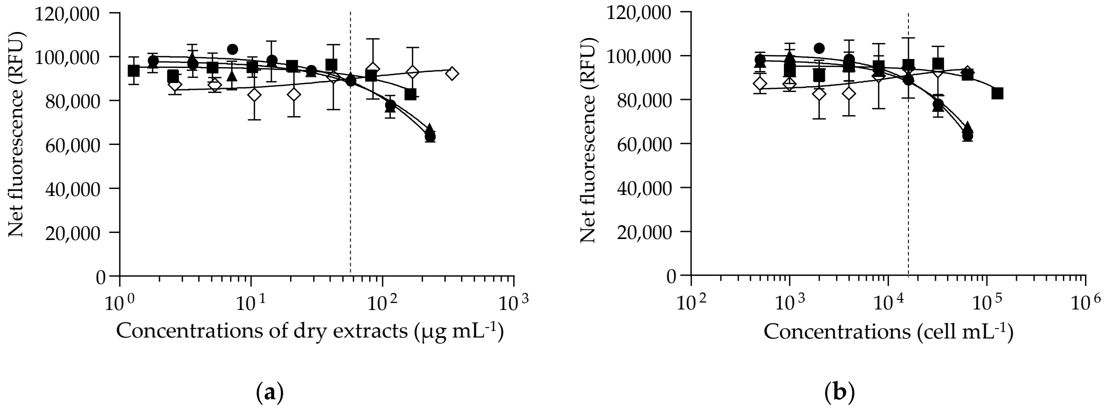

Figure S2. Binding dose-response curves of *Gambierdiscus pacificus (*TKR9 ■) and *G. toxicus* (RIK31●, HPT3 ◊, and TKU5 ▲) samples using the fluorescent receptor binding assay (fRBA) with incubation conditions tested at 37°C during 30 min. Matrix effects of four *Gambierdiscus* strains with their concentrations expressed (a) in μg of dry extract mL^{-1} and (b) in cell mL^{-1}. Data represent the mean ± standard deviation (SD) of one experiment, each concentration run in duplicate wells. The dotted vertical lines correspond to the maximum concentration of *Gambierdiscus* extract equivalent to MCE = 55 ± 26 μg of dry extract mL^{-1} corresponding to 16,000 cell mL^{-1} for matrix interference.
